# Activation of Kir4.1 Channels by 2‐D08 Promotes Myelin Repair in Multiple Sclerosis

**DOI:** 10.1002/advs.202502032

**Published:** 2025-06-05

**Authors:** Mingdong Liu, Shengyu Jin, Xin Fu, Chong Xie, Yi Chen, Liangtang Chang, Yongheng Fan, Donghua He, Xiaoqi Hong, Xi Shen, Xiaoli Zheng, Qiyue Wang, Dao Shi, Fangyuan Li, Daishun Ling, Yangtai Guan, Neng Gong, Xiaoping Tong

**Affiliations:** ^1^ Department of Obstetrics and Gynecology Songjiang Research Institute Shanghai Key Laboratory of Emotions and Affective Disorders Songjiang Hospital Affiliated to Shanghai Jiao Tong University School of Medicine Shanghai 201600 China; ^2^ Department of Anatomy and Physiology Shanghai Jiao Tong University School of Medicine Shanghai 200025 China; ^3^ Center for Brain Science Shanghai Children's Medical Center Shanghai Jiao Tong University School of Medicine Shanghai 200127 China; ^4^ Department of Neurology Ren Ji Hospital Shanghai Jiao Tong University School of Medicine Shanghai 200127 China; ^5^ Institute of Neuroscience Center for Excellence in Brain Science and Intelligence Technology Chinese Academy of Sciences Shanghai 200031 China; ^6^ Frontiers Science Center for Transformative Molecules School of Chemistry and Chemical Engineering School of Biomedical Engineering National Center for Translational Medicine Shanghai Jiao Tong University Shanghai 200240 China; ^7^ Songjiang Research Institute Shanghai Key Laboratory of Emotions and Affective Disorders Shanghai Jiao Tong University School of Medicine Shanghai 201600 China; ^8^ Department of Neurology, Punan Branch of Renji Hospital Shanghai Jiaotong University School of Medicine Shanghai 200125 China

**Keywords:** 2‐D08, demyelinating diseases, Kir4.1, multiple sclerosis, remyelination

## Abstract

Multiple sclerosis (MS) is a chronic inflammatory disease that leads to myelin loss and neurological dysfunction. Clinical studies show increased anti‐Kir4.1 antibody levels in MS patients' serum, indicating its diagnostic potential. However, the specific mechanism has remained elusive. In a mouse model of experimental autoimmune encephalomyelitis (EAE), it is found that impaired Kir4.1 channels in oligodendrocyte precursor cells (OPCs) hindered myelin repair in the spinal cord. Using a thermal shift assay (TSA), the small molecule 2‐D08 is identified, which effectively activated Kir4.1 channels and reduced demyelination in both EAE mice and marmosets. The neuroprotective effects are mainly due to enhanced phosphorylation of FYN tyrosine kinase, promoting OPCs differentiation. The findings highlight the critical role of Kir4.1 channels in MS pathogenesis and suggest that pharmacological activation of these channels by 2‐D08 can be a promising therapeutic strategy for enhancing brain recovery in demyelinating diseases.

## Graphical abstract

Multiple sclerosis causes myelin loss and neurological dysfunction. This study shows that 2‐D08, a small molecule targeting Kir4.1 channels, promotes OPCs differentiation via FYN tyrosine kinase phosphorylation and the FYN/MYRF pathway. It significantly improves myelin repair and motor deficits in EAE mice and marmosets, highlighting its potential as a therapy for demyelinating diseases.

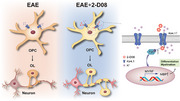

## 1 Introduction

Multiple sclerosis (MS) is a complex disease characterized by demyelination in various areas of the central nervous system (CNS). Considered as an autoimmune disorder, MS involves inflammation, demyelination, and neurodegeneration, leading to significant neurological disability.^[^ ^1^ ^]^ Initially, MS presents as relapses and remissions, but later progresses into a secondary progressive phase without periods of remission. This chronic condition predominantly affects young individuals, impacting their white matter, spinal cord, optic nerve, and resulting in debilitating neurological symptoms such as limb numbness, ataxia, cognitive impairment, and depression.^[^ ^2^, ^3^ ^]^ Unfortunately, there is currently no effective treatment to cure MS, highlighting the urgent need for new therapeutic strategies.

One of the significant advancements in MS research is the identification of the potassium channel Kir4.1 as an autoimmune target, offering new possibilities for expanding the therapeutic options for MS.^[^ ^4^, ^5^ ^]^ In 2012, Srivastava and colleagues reported higher levels of autoantibodies against the Kir4.1 channel in the serum of MS patients,^[^ ^5^ ^]^ which have been subsequently confirmed by numerous studies as well.^[^ ^6^, ^7^, ^8^ ^]^ However, the cause of increased serum Kir4.1 antibodies and their relationship to MS pathogenesis is currently inconclusive.^[^ ^9^, ^10^ ^]^ Kir channels, including Kir4.1, are a type of inwardly rectifying potassium channel that facilitate the entry of K^+^ ions into cells rather than their efflux. Kir4.1 channels are widely expressed in various glial cell types, such as astrocytes and oligodendrocyte lineage cells.^[^ ^11^, ^12^, ^13^, ^14^ ^]^ They play crucial roles in maintaining the resting membrane potential (RMP), regulating extracellular K^+^ uptake, sensing local K^+^ levels, controlling cell volume, facilitating glutamate uptake, and are also implicated in various brain disorders like Huntington's disease, white matter injury, and ischemia.^[^ ^11^, ^12^, ^13^, ^14^, ^15^, ^16^, ^17^, ^18^ ^]^

Among the therapeutic approaches targeting ion channels for the treatment of MS, dalfampridine has been reported to be highly effective in improving the walking ability of MS patients.^[^ ^19^, ^20^ ^]^ Dalfampridine as a sustained‐release form of 4‐aminopyridine (4‐AP), is sensitive to voltage‐gated potassium (Kv) channels. Studies have shown that dalfampridine, by blocking neuronal Kv channels, can enhance conduction along demyelinated axons, resulting in improvements in visual function, motor skills, and relief from fatigue in MS patients. As a result, it is currently being used as a clinical drug for the symptomatic treatment of MS.^[^ ^21^ ^]^ However, there is increasing evidence indicating that higher doses of dalfampridine may increase the risk of seizures in MS patients. This is because 4‐AP, the active ingredient in dalfampridine, has been shown to induce acute epileptic seizures in numerous animal studies.^[^ ^22^, ^23^, ^24^, ^25^, ^26^, ^27^ ^]^ Therefore, the FDA advises against prescribing this drug to MS patients with a current or previous history of seizures, moderate or severe renal impairment, and a history of allergy to AMPYRA or 4‐aminopyridine, limiting the treatment's benefits for a broader patient population.

The Kir4.1 channel, a subtype of potassium channels, has been proposed as a therapeutic target in MS. However, the development of pharmaceutical drugs targeting this channel for potential MS treatment lags far behind the rapid clinical progress of dalfampridine, which focuses on Kv channels. This delay can be attributed, in large part, to the uncertainty surrounding the pathogenic nature of the Kir4.1 autoantibody. It remains unclear whether the autoantibody is a byproduct of the MS degenerative process or a contributing factor. Some even argue that blocking increased Kir4.1 activity could potentially lead to severe epilepsy, ataxia, and deafness in humans.^[^ ^28^ ^]^ Interestingly, a very recent study suggests that a dysconnection in K^+^ shuttling between axonal Kv7 and oligodendroglial Kir4.1 contributes to the pathogenesis of MS, as evidenced by MS‐like symptoms observed in Kir4.1‐deficient mice.^[^ ^29^ ^]^ Therefore, the activation or inhibition of Kir4.1 activity remains a widely challenging aspect of strategic MS treatment. Investigating and clarifying the pathogenic role of Kir4.1 is imperative to explore new therapeutic approaches that can benefit a larger proportion of MS patients.

In our current study, we made an intriguing discovery that an initial impairment of Kir4.1 channels occurred in spinal cord oligodendrocyte precursor cells (OPCs) rather than in astrocytes in a mouse model of experimental autoimmune encephalomyelitis (EAE), while the levels of Kir4.1 antibodies in both EAE mouse serum and MS patient serum were significantly elevated. Through a thermal shift assay (TSA) screening process, we successfully identified a small molecule compound called 2‐D08 as a potent activator of the Kir4.1 channel. By treating EAE mice with 2‐D08, EAE‐induced demyelination was significantly alleviated in the spinal cord, leading to noticeable improvements in motor function as demonstrated by grid walking and inclined plane test in EAE mice. The underlying mechanism primarily involves enhanced phosphorylation of FYN tyrosine kinase, which results from the activation of the Kir4.1 channel. This, in turn, enhances FYN/myelin regulatory factor (MYRF) signaling to promote oligodendrocyte maturation and remyelination.^[^ ^30^ ^]^ Importantly, the compound 2‐D08 has demonstrated brain protection effects similar to dalfampridine in promoting myelin repair and motor recovery in both EAE mice and marmosets, without inducing seizure‐like epileptic activity. Therefore, our study emphasizes the potential of 2‐D08 to have broader clinical and pharmaceutical applications, ultimately benefiting a larger population of MS patients.

## 2 Results

### 2.1 Impairment of the Kir4.1 Ion Channel in OPCs Leads to the Failure of Myelin Repair in the Ventral Spinal Cord of EAE Mice

**Figure 1:**
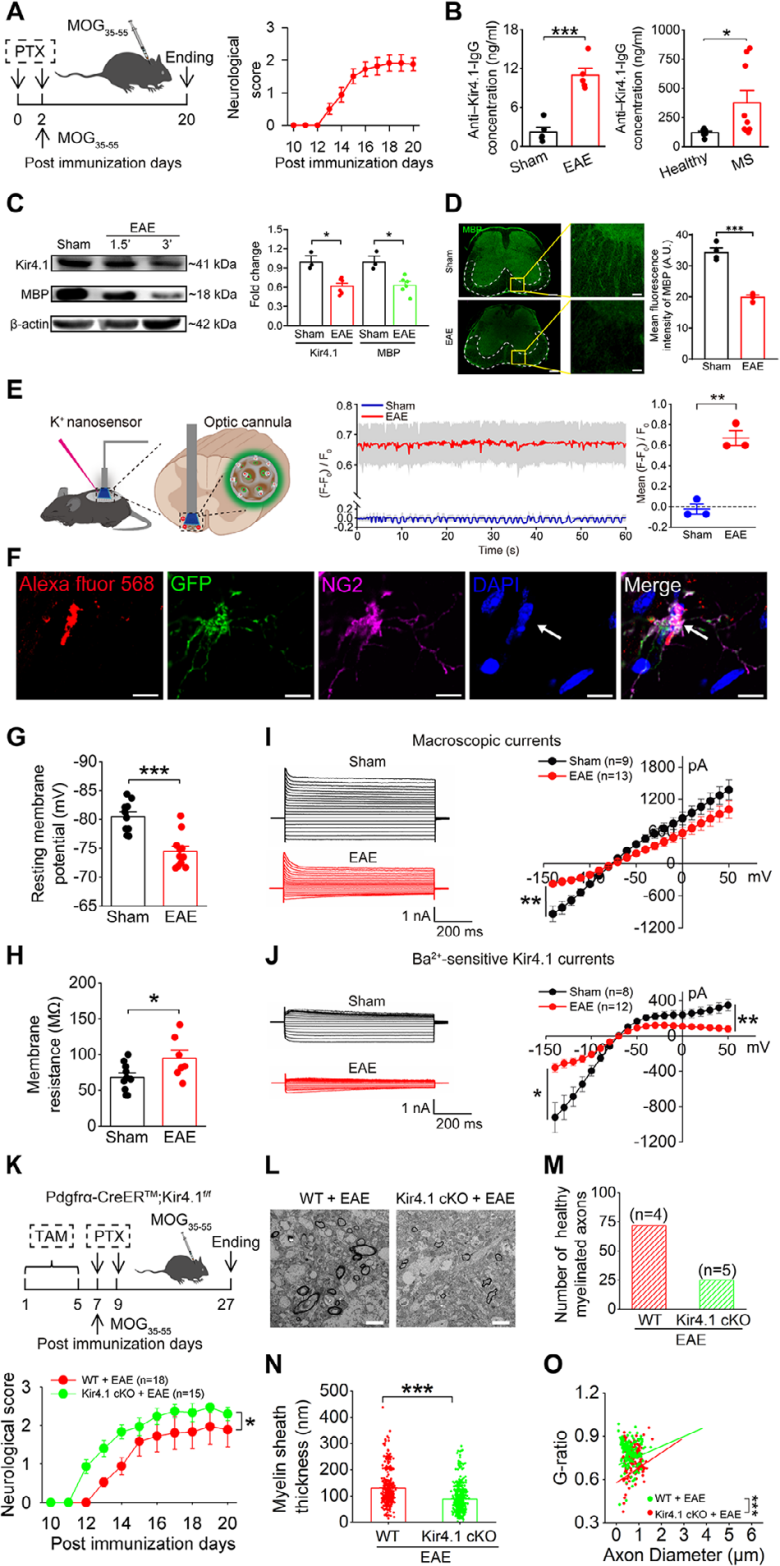
Impairment of Kir4.1 ion channels in OPCs and demyelination in ventral spinal cord of EAE mice. A,B) EAE mouse model induction (A) and the concentration of Kir4.1 antibody in the serums of EAE mice and MS patients (B). For mice analysis, n = 5 and 5 mice for sham and EAE groups, respectively, two‐tailed unpaired t‐test, ***p < 0.001. For human samples analysis, n = 6 for healthy individuals and n = 9 for MS patients, two‐tailed Mann‐Whitney test, *p < 0.05. C) Representative images of protein levels of Kir4.1 and MBP in sham and EAE mice (EAE scores at 1.5 and 3). Bar graph showed the quantitative statistics of sham and EAE mice, n = 3 mice for sham and n = 6 mice for EAE. For Kir4.1 analysis, two‐tailed Mann–Whitney test, *p < 0.05. For MBP analysis, two‐tailed Mann–Whitney test, *p < 0.05. D) The left panel showed sample images of MBP immunostaining within the spinal cords of sham and EAE mice. The right panels displayed magnified views of the MBP immunostaining in the areas indicated by the yellow squares. Scale bars: 200 µm (left panel) and 50 µm (right panels). The data points in the graph represented the number of mice analyzed, n = 4 and 4 mice for sham and EAE, respectively, two‐tailed unpaired t‐test, ***p < 0.001. E) Schematic diagram showed optical [K^+^]_o_ sensing in ventral spinal cord and both representative trace and bar graph showed fluorescence responses of the K^+^ ion sensors to the EAE and sham mice, n = 3 mice for each group, two‐tailed unpaired t‐test, **p < 0.01. F) Whole‐cell patched OPCs expressing GFP loaded with Alexa Fluor 568 in the patch pipette and identified with post‐immunostaining of NG2 antibody. Scale bars: 10 µm. (G‐J) Electrophysiological changes of RMPs (G), membrane resistances (H), macroscopic K^+^ currents (I) and Ba^2+^‐sensitive Kir4.1 currents (J) from 4 sham mice and 5 EAE mice, respectively. For RMP analysis, n = 10 cells and 11 cells from sham and EAE mice, respectively, two‐tailed unpaired t‐test, ***p < 0.001. For membrane resistance analysis, n = 8 cells and 8 cells from sham and EAE mice, two‐tailed Mann–Whitney test, *p < 0.05. For macroscopic K^+^ currents analysis, n = 9 cells and 13 cells from sham and EAE mice, respectively, two‐tailed Mann–Whitney test, **p < 0.01 at a holding voltage of −140 mV. For Kir4.1 currents analysis, n = 8 cells and 12 cells from sham and EAE mice, respectively, two‐tailed Mann–Whitney test, *p < 0.05 at a holding voltage of −140 mV. K) Schematic diagram showing EAE disease model in Pdgfrα‐creER^TM^; Kir4.1^f/f^ mice to specifically knock down Kir4.1 expression in OPCs (upper panel) and neurological scores of Kir4.1 cKO mice and WT mice were determined after EAE induction (lower panel). n = 15 mice and 18 mice from Kir4.1 cKO and WT mice, respectively, two‐tailed Mann–Whitney test, *p < 0.05. L,M) Representative electron micrographs (L) and bar graph summarized the numbers of healthy myelinated axons (myelin sheaths thickness > 200 nm) (M) between WT mice and Kir4.1 cKO mice after EAE, n = 4 mice and 5 mice from WT mice and Kir4.1 cKO, respectively. Scale bars, 2 µm. N,O) Box‐plots and G ratio of myelinated axons illustrated the average of myelin sheath thickness (N) and G‐ratio (O) between WT mice and Kir4.1 cKO mice after EAE. n = 4 mice and 5 mice from WT mice and Kir4.1 cKO, respectively. For myelin sheath thickness analysis, two‐tailed Mann–Whitney test, ***p < 0.001. For G‐ratio of myelinated axons analysis, simple linear regression of slopes, ***p < 0.001.

Initially, an EAE mouse model was induced by immunizing mice with the MOG_35‐55_ peptide (**Figure** **1**A). Disease progression was evaluated through neurobehavioral analysis, using a well‐established symptom scale.^[^ ^31^ ^]^ The first signs of EAE induction in mice were observed at 13–14 days and reached their peak at 18–20 days after MOG_35‐55_ injections (Figure 1A). Considering prior findings that increased serum Kir4.1 antibody levels are associated with MS patients,^[^ ^32^ ^]^ we utilized ELISA to detect the expression level of anti‐Kir4.1 antibodies in serum samples obtained from both EAE mice and MS patients (Figure 1B; Table S1, Supporting Information). The results indicated that IgG reactivity against Kir4.1 protein was significantly elevated in EAE mice, as well as in MS patients (Figure 1B). Next, we conducted western blotting analysis to examine MBP protein expression in the spine cord of EAE mice, as axonal demyelination in the EAE model predominantly occurs in the ventral regions of the spinal cord.^[^ ^31^, ^33^ ^]^ Compared to sham mice, we observed a significant reduction in MBP protein levels in the spinal cords of mice with an EAE score of 3 (Figure 1C). Moreover, MBP and SMI32 immunohistochemistry, as well as gold myelin staining, revealed severe myelin loss in the spinal cords of EAE mice (Figure 1D; Figure S1, Supporting Information), indicating that pronounced demyelination had occurred in these mice. Additionally, we found that Kir4.1 protein expression in the spinal cord was also substantially reduced in EAE mice compared to sham mice (Figure 1C).

Since the deficits of Kir4.1 could lead to an elevated K^+^ level in extracellular space due to the decline in buffering potassium ion capacity, we next performed in vivo potassium ion measurements by K^+^ nanosensor.^[^ ^18^, ^34^ ^]^ The fluorescence intensity of the K^+^ probe revealed a significant elevation in extracellular K^+^ levels in the spinal cord of EAE mice compared to that in sham‐operated mice (Figure 1E). To delve deeper into the alterations in Kir4.1 channel activity throughout the progression of EAE, we conducted a systematic analysis of Kir4.1 channel activities in both astrocytes and OPCs from EAE and sham mice, as Kir4.1 ion channels are primarily expressed in astrocyte and oligodendrocyte lineage cells.^[^ ^18^, ^35^, ^36^ ^]^ Previous studies have reported a significant accumulation of quiescent OPCs at lesions in MS, along with increased cell death of oligodendrocytes during neuroinflammation.^[^ ^37^, ^38^ ^]^ In light of this, we conducted electrophysiological recordings of OPCs from acute lumbar ventral spinal cord slices obtained from Pdgfrα‐creER^TM^; mGFP mice. The immunohistochemistry results demonstrated that 96.84% of the GFP‐expressing cells were colocalized with anti‐Pdgfrα antibody‐labeled OPCs in the spinal cord (Figure S2, Supporting Information). Furthermore, as shown in Figure 1F, single patch‐recorded GFP‐positive cells from Pdgfrα‐creER^TM^; mGFP mice were post‐immunostained and displayed clear colocalization with the NG2 antibody. Our electrophysiological results showed a significant decrease in both macroscopic K^+^channel currents and Ba^2+^‐sensitive Kir4.1 channel currents, as well as impaired membrane properties, in ventral OPCs compared to those in dorsal OPCs of EAE mice (Figure 1G–J; Figure S3, Supporting Information). In contrast, neither membrane properties nor Kir4.1 channel activity were altered in ventral spinal cord astrocytes of EAE mice (Figure S4, Supporting Information). Taken together, these results indicate that both impairments of axonal myelin sheaths and deficits of Kir4.1 channels in ventral spinal cord OPCs initially occur in EAE mice.

To further investigate the correlation between Kir4.1 impairment and demyelination in EAE mice, we utilized Kir4.1 conditional knockout (cKO) mice to specifically reduce the expression of *kcnj10*‐encoded Kir4.1 channels in OPCs (Pdgfrα‐creER^TM^; Kir4.1^f/f^). The knockdown efficiency was confirmed through single‐cell RT‐PCR analysis (Figure S5A, Supporting Information). We found that EAE scores were significantly worse in Kir4.1 cKO mice compared to wild‐type (WT) mice following MOG_35‐55_ immunization (Figure 1K). However, the knockout of Kir4.1 did not affect the presence of mature oligodendrocytes (Figure S5B, Supporting Information) or basic physiological and behavioral functions after myelin formation in adult mice (Figure S5C–F, Supporting Information). Electron microscopy imaging further demonstrated the direct impact of the Kir4.1 channel in OPCs on myelin loss in Kir4.1‐deficient mice after EAE (Figure 1L–O). Specifically, healthy axons with myelin sheath thickness greater than 200 nm (Figure 1M), total myelin sheath thickness (Figure 1N) and the G‐ratio (Figure 1O) in the ventral spinal cord were significantly impaired in Kir4.1 cKO mice compared to WT mice after EAE. In contrast, when we specifically knocked down Kir4.1 in astrocytes of Kir4.1^f/f^ mice using AAV2/5‐gfaABC1D‐ERT2‐Cre‐ERT2‐WPRE‐pA viral injections, we observed no further deterioration of motor functions in Kir4.1 cKO mice after EAE (Figure S6, Supporting Information). In summary, these findings suggest that the impairment of Kir4.1 channels expressed in OPCs resulted in more severe pathological symptoms in EAE mice.

### 2.2 The 2‐D08 Compound Binds to the Kir4.1 Protein and Augments the Kir4.1 Channel Activity in OPCs

**Figure 2:**
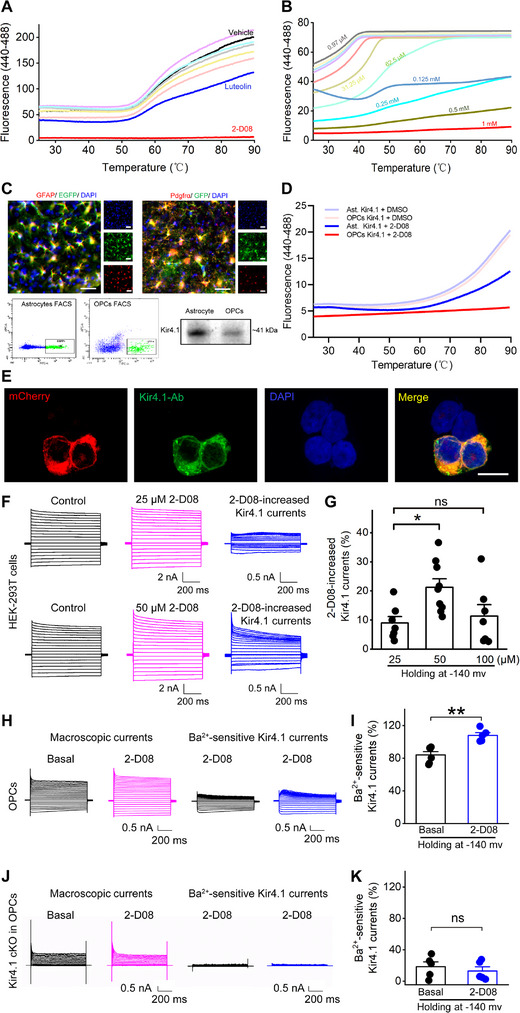
2‐D08 bound to the Kir4.1 protein and augmented the Kir4.1 channel activity. A,B) Typical melting curves were obtained from six structural analogues of luteolin, monitoring the thermal denaturation of Kir4.1 protein with increasing temperature (A) and 2‐D08 binding effect with Kir4.1 ranging from 0.97 µm to 1 mm (B). C) The upper panel displayed GFP‐labeled astrocytes or OPCs obtained from rAAV2/PHP.eB‐GFAP‐EGFP virus‐transfected WT mice or Pdgfrα‐creER^TM^; mGFP transgenic mice (n = 3 for each group). The lower panel represented EGFP^+^ astrocytes or GFP^+^ OPCs collected by FACS and purified Kir4.1 protein was extracted from isolated astrocytes or OPCs with immunoprecipitation method. D) TSA analysis revealed the 2‐D08 binding effect with Kir4.1 from flow cytometry sorted astrocytes and OPCs. E) Representative immunohistochemistry images showed that Kir4.1‐Ab labeling (green) was specifically expressed in Kir4.1‐mCherry transfected HEK‐293T cells (red). Scale bar, 10 µm. F,G) Representative traces (F) and bar graph summary (G) showed that different doses of 2‐D08 induced an increase in Kir4.1 currents in Kir4.1‐mCherry transfected HEK‐293T cells. n = 7 cells for 25 and 100 µm, and n = 9 cells for 50 µm from three independent experiments, one‐way ANOVA with Tukey–Kramer multiple comparisons test, *p < 0.05. H–K) Representative traces and bar graphs summary showed the effect of 2‐D08 in Kir4.1 currents in OPCs from WT [(H) and (I)] (n = 6 cells for basal and n = 5 cells for 2‐D08 group, two‐tailed unpaired t‐test, **p < 0.01) and cKO mice [(J and K)] (n = 5 cells for each basal and 2‐D08 group, two‐tailed unpaired t‐test, *p* = 0.5172).

Given our discovery of impaired Kir4.1 channel activity in OPCs of EAE mice and its significant contribution to the failure of myelin repair in the EAE disease model, we sought to identify a potential activator targeting the Kir4.1 channel to promote myelin repair. To assess protein–ligand interactions, we utilized a fluorescence‐based biochemical technique known as the TSA, a well‐established method for detecting protein thermal stability.^[^ ^39^ ^]^ We first conducted melt curve experiments using six structural analogues of luteolin (Figure S7, Supporting Information), a competitive compound reported in our previous study to promote myelin repair by targeting K^+^ channels.^[^ ^35^ ^]^ Of these six molecules, we observed that 2‐D08 exhibited a stronger targeting effect on the Kir4.1 protein than luteolin (**Figure** **2**A). Furthermore, 2‐D08 induced a concentration‐dependent increase in the melting temperature, ranging from 0.97 µm to 1 mm (Figure 2B), suggesting a positive correlation between the binding interaction of 2‐D08 and the Kir4.1 protein. The Kir4.1 channel is expressed not only in OPCs but also in astrocytes. To investigate the cell type targeted by 2‐D08, we labeled astrocytes through intravenous injection of the rAAV2/PHP.eB‐GFAP‐EGFP virus in C57BL/6J mice and obtained OPCs from Pdgfrα‐creER^TM^; mGFP transgenic mice. We then isolated astrocytes and OPCs from these mice using fluorescence‐ activated cell sorting (FACS) based on GFP expression and assessed Kir4.1 protein expression in the harvested cells (Figure 2C). These data confirmed that both astrocytes and OPCs express Kir4.1. Furthermore, TSA screening analysis revealed that 2‐D08 displayed a strong affinity with the Kir4.1 protein expressed in OPCs but exhibited weak interaction with the Kir4.1 protein expressed in astrocytes (Figure 2D), indicating that 2‐D08 primarily exerts pharmacological effects by targeting Kir4.1 in OPCs. To determine whether 2‐D08 functions as an inhibitor or activator of Kir4.1 channel activity, we first transfected Kir4.1 plasmids (Plvx‐Kcnj10‐IRES‐mCherry) into HEK‐293T cells and employed a rapid drug delivery system to measure Kir4.1 channel currents at different concentrations of 2‐D08 during application (Figure 2E). Among these concentrations, 50 µM 2‐D08 elicited the maximum net increase in Kir4.1 channel currents in transfected HEK‐293T cells when the cell voltage was held at −140 mV (Figure 2F,G). To directly investigate the impact of 2‐D08 on Kir4.1 channels in OPCs, which contribute significantly to inward K^+^ channel currents (82.2% at a holding voltage of −140 mV),^[^ ^14^ ^]^ we assessed the net increase of Ba^2+^‐sensitive Kir4.1 current in ventral spinal cord OPCs. The results demonstrated a 27.1% net increase in Kir4.1 currents upon application of 50 µm 2‐D08 compared to the basal condition when the cell voltage was held at −140 mV (Figure 2H,I). Moreover, 2‐D08 failed to induce an augmentation of Kir4.1 channel currents in Kir4.1‐deficient OPCs (Figure 2J,K). In contrast, 2‐D08 did not affect Ba^2+^‐sensitive Kir4.1 current in astrocytes, nor did it alter the electrophysiological membrane properties of these cells (Figure S8, Supporting Information).

Given the presence of Kir4.1 autoantibodies in both EAE mice and MS patient sera, we investigated whether Kir4.1 antibodies might interfere with 2‐D08‐mediated activation of Kir4.1 channels. We transfected HEK‐293T cells with a Kir4.1 plasmid and assessed channel activity following exposure to anti‐Kir4.1 antibodies. Patch‐clamp recordings revealed that incubation with Kir4.1 antibodies in the recording buffer did not significantly alter 2‐D08‐induced Kir4.1 channel currents (Figure S9, Supporting Information). In parallel experiments using primary cultured OPCs, we observed that Kir4.1 and MBP expression were upregulated during OPC differentiation into oligodendrocytes (Figure S10, Supporting Information). Although 50 µm 2‐D08 successfully enhanced OPC differentiation (Figure S11A, Supporting Information), neither Kir4.1 nor MBP protein expression was altered in mature oligodendrocytes cultured with 2‐D08 treatment (Figure S11B, Supporting Information). Collectively, these findings demonstrate that 2‐D08 specifically binds to and activates Kir4.1 channels in OPCs without affecting astrocytes or mature oligodendrocytes.

### 2.3 Administration of 2‐D08 Promotes Myelin Repair in Spinal Cord of EAE Mice

**Figure 3:**
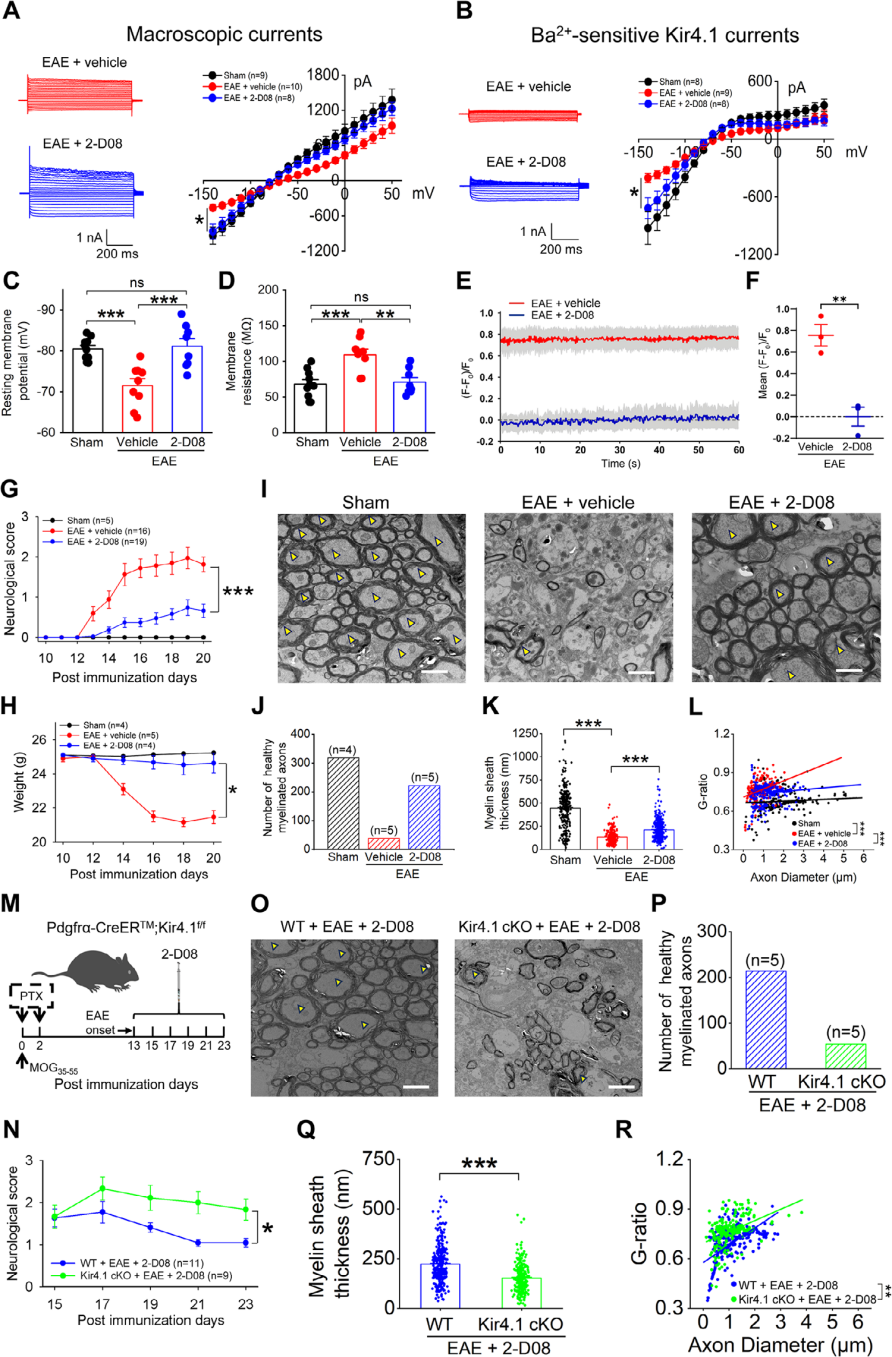
Administration of 2‐D08 promoted myelin repair in spinal cord of EAE mice. A–D) Macroscopic K^+^ currents (A), Ba^2+^‐sensitive Kir4.1 currents (B), RMPs (C) and membrane resistances (D) in ventral spinal cord OPCs in sham, EAE + vehicle and EAE + 2‐D08 mice, respectively. For macroscopic K^+^ currents analysis, n = 9 cells, 10 cells and 8 cells for sham, EAE + vehicle and EAE + 2‐D08 group, respectively, two‐tailed Mann–Whitney test, *p < 0.05 between the EAE + vehicle and EAE + 2‐D08 group at the cell holding voltage of −140 mV. For Kir4.1 currents analysis, n = 8 cells, 9 cells and 8 cells for sham, EAE + vehicle and EAE + 2‐D08, respectively, two‐tailed Mann–Whitney test, *p < 0.05 between the EAE + vehicle and EAE + 2‐D08 group at the cell holding voltage of −140 mV. For RMP analysis, n = 10 cells, 9 cells and 8 cells for sham, EAE + vehicle and EAE + 2‐D08, respectively, one‐way ANOVA with Tukey–Kramer multiple comparisons test, ***p < 0.001, ns: not significant. For membrane resistance analysis, n = 10 cells, 8 cells and 8 cells for sham, EAE + vehicle and EAE + 2‐D08, respectively, one‐way ANOVA with Tukey–Kramer multiple comparisons test, **p < 0.01, ***p < 0.001, ns: not significant. E,F) Representative trace (E) and bar graph (F) showed fluorescence responses of the K^+^ ion sensors to EAE + vehicle and EAE + 2‐D08 group mice, n = 3 mice for each group, two‐tailed unpaired t‐test, **p < 0.01. G,H) 2‐D08 ameliorated EAE neurological scores (G) (n = 16 mice and 19 mice for the EAE + vehicle and EAE + 2‐D08 groups, respectively, one‐way ANOVA with Tukey‐Kramer multiple comparisons test, ***p < 0.001) and improved weights recovery (H) (n = 5 mice and 4 mice for the EAE + vehicle and EAE + 2‐D08 groups, respectively, one‐way ANOVA with Tukey–Kramer multiple comparisons test, *p < 0.05) compared to vehicle treated EAE mice. I–L) Electron microscopy was performed on axons from sham, EAE + vehicle and EAE + 2‐D08 mice. Representative electron micrographs showed healthy myelin sheaths (I), the number of healthy myelinated axons (myelin sheaths thickness > 200 nm) (J), myelin sheath thickness (K) and G‐ratio (L) under different conditions. The yellow triangles highlight samples of healthy myelinated axons with myelin sheaths greater than 200 nm. Scale bars, 2 µm. n = 4, 5, 5 mice for the sham, EAE + vehicle and EAE + 2‐D08 groups, respectively. For myelin sheath thickness analysis, one‐way ANOVA with Kruskal–Wallis multiple comparisons test, ***p < 0.001. For G‐ratio of myelinated axons analysis, simple linear regression of slopes, ***p < 0.001, Kruskal–Wallis with Dunn's multiple comparisons test. M,N) Experimental diagram in Kir4.1 cKO EAE mice treated with 2‐D08 starting on disease onset after MOG_35‐55_ immunization (M) and the neurological scores (N) in both WT and Kir4.1 cKO mice after treatment. n = 11 mice and 9 mice for the WT and Kir4.1 cKO groups, respectively, two‐tailed unpaired t‐test, *p < 0.05. O–R) Electron microscopy analyzed axons in the presence of 2‐D08 following EAE in both WT and Kir4.1 cKO mice. Representative electron micrographs, the yellow triangles mark healthy myelin sheaths (O), the number of healthy myelinated axons (myelin sheaths thickness > 200 nm) (P), myelin sheath thickness (Q) and G‐ratio (R). Scale bars, 2 µm. n = 5 mice for each group. For myelin sheath thickness analysis, two‐tailed unpaired t‐test, ***p < 0.001. For G‐ratio of myelinated axons analysis, simple linear regression of slopes, **p < 0.01.

We next investigated the direct impact of 2‐D08 on myelination and its potential in promoting myelin repair in EAE mice by targeting Kir4.1 channels. The mice received daily intraperitoneal injections of either 2‐D08 (1 mg kg^−1^) or vehicle, starting 48 h after MOG_35‐55_ immunization. Pharmacokinetic analysis revealed that due to its small molecular weight of 270, 2‐D08 exhibited excellent blood–brain barrier penetration and rapid enrichment in brain tissue (Figure S12, Supporting Information). Electrophysiological recordings from acute lumbar ventral spinal cord slices revealed a notable increase in both macroscopic K^+^ channel currents and Ba^2+^‐sensitive Kir4.1 channel currents, along with restored membrane properties in OPCs from EAE mice treated with 2‐D08, compared to those treated with vehicle (**Figure** **3**A–D). Additionally, we observed that the enhanced fluorescence intensity of the K^+^ nanosensor was significantly decreased after 2‐D08 administration in EAE mice (Figure 3E,F), indicating that the elevated extracellular K^+^ levels returned to normal following the restoration of Kir4.1 channel activity in OPCs.

Based on this evidence, we further assessed the effectiveness of myelin repair following 2‐D08 treatment 48 h after MOG_35‐55_ immunization. Neurological scores and overall body weight indicated that 2‐D08 significantly alleviated disease severity and promoted weight gain during the acute phase of EAE in mice (Figure 3G,H). The 2‐D08‐treated group exhibited enhanced myelin repair compared to the vehicle‐treated EAE mice, as evidenced by increased gold myelin staining, elevated MBP levels, and reduced SMI32 expression (Figure S13, Supporting Information). Notably, electron microscopy analysis further revealed an increased number of healthy myelinated axons (with myelin sheath thickness greater than 200 nm), thicker myelin sheaths, and a lower G‐ratio in 2‐D08‐treated mice (Figure 3I–L). However, clinical EAE scores were worse in Kir4.1 cKO mice compared to WT EAE mice after 2‐D08 treatment (Figure 3M,N). The number of healthy myelinated axons was decreased, and the myelin repair process was hindered in Kir4.1 cKO mice compared to WT mice when both groups were treated with 2‐D08 after EAE (Figure 3O–R).

To further investigate the potential of myelin repair by 2‐D08, we used a lysolecithin (LPC)‐induced focal demyelination mouse model, which is independent of immune‐mediated mechanisms (Figure S14A, Supporting Information).^[^ ^2^, ^40^ ^]^ We observed that 2‐D08 treatment significantly promoted myelin repair in the spinal cord at 7 and 14 days post‐lesion (dpl) following LPC injections. Additionally, 2‐D08 treatment substantially increased MBP levels while reducing SMI32 expressions in the spinal cord lesion areas (Figure S14B,C, Supporting Information). Taken together, these results demonstrate that 2‐D08 effectively promotes myelin repair by activating Kir4.1 channels in OPCs, thereby highlighting its potential across various demyelinating contexts.

### 2.4 2‐D08 Treatment Improves Movement Capability and Neurological Functional Recovery in EAE Mice

**Figure 4:**
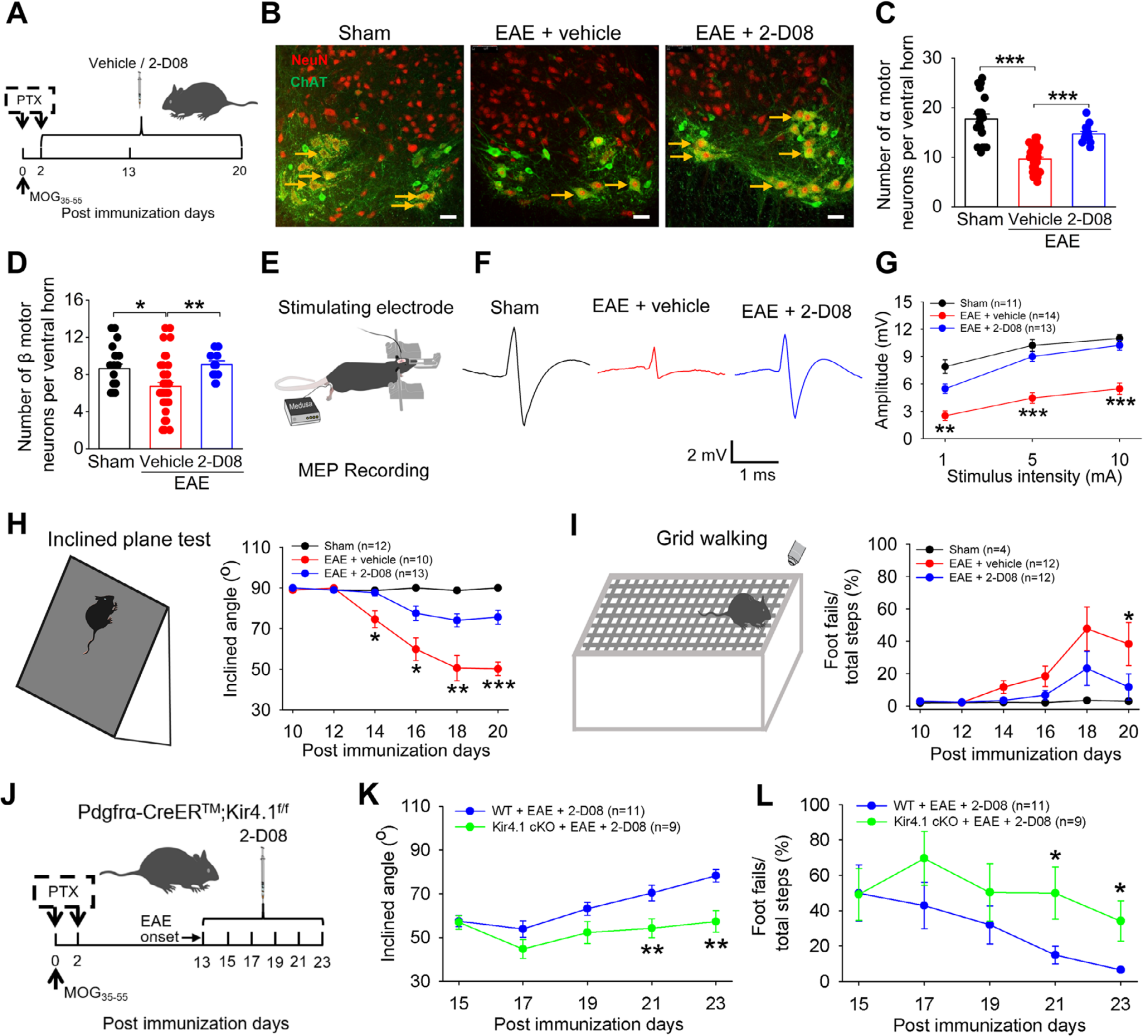
2‐D08 treatment improved movement capability and neurological functional recovery in EAE mice. A) The cartoon illustrated the experimental scheme in which EAE mice were treated with vehicle or 2‐D08 (i.p.) 48 hours after immunization with MOG_35‐55_. B–D) Representative immunohistochemical images (B), and summary bar graphs of α motor neurons (C) and β motor neurons (D) in the spinal cord ventral horn of sham mice, EAE + vehicle mice, and EAE + 2‐D08 mice, respectively. For the number of α motor neurons analysis, n = 4, 3, and 3 mice for the sham, EAE + vehicle, and EAE + 2‐D08 groups, respectively, one‐way ANOVA with Kruskal–Wallis multiple comparisons test, ***p < 0.001. For the number of β motor neurons analysis, n = 4, 3 and 3 mice for the sham, EAE + vehicle, and EAE + 2‐D08 groups, respectively, one‐way ANOVA with Kruskal–Wallis multiple comparisons test, *p < 0.05, **p < 0.01. Scale bars: 50 µm. E–G) Schematic cartoon illustrated the experimental diagram for measuring MEPs (E), representative sample recordings (F) and comparison of MEP amplitudes from sham, EAE + vehicle and EAE + 2‐D08 treated mice (G). n = 11, 14 and 13 mice for sham, EAE + vehicle and EAE + 2‐D08 groups, respectively, one‐way ANOVA with Tukey–Kramer multiple comparisons test, **p < 0.01 between the EAE + vehicle and EAE + 2‐D08 treated group, ***p < 0.001 between the EAE + vehicle and EAE + 2‐D08 treated group. H) Schematic cartoon of the inclined plane test (left panel) and the summary graph (right panel) showed that EAE mice under the treatment with 2‐D08. n = 10 mice for the EAE + vehicle group and n = 13 mice for EAE + 2‐D08 group, two‐tailed unpaired t‐test, *p < 0.05 between the EAE + vehicle and EAE + 2‐D08 treated group, **p < 0.01 between the EAE + vehicle and EAE + 2‐D08 treated group, ***p < 0.001 between the EAE + vehicle and EAE + 2‐D08 treated group. I) Schematic cartoon illustrated the motor ability of mice in the grid walking task (left panel) and summary graph (right panel) showed that EAE mice under the treatment with 2‐D08. n = 12 mice for EAE + vehicle group and n = 12 mice for EAE + 2‐D08 group, respectively, two‐tailed unpaired t‐test, *p < 0.05 between the EAE + vehicle and EAE + 2‐D08 treated group. J) The cartoon illustrated the experimental diagram in Kir4.1 cKO mice treated with 2‐D08 (i.p.) starting on day 13 (disease onset) after MOG_35‐55_ immunization. K) Inclined angle of WT mice and Kir4.1 cKO mice with 2‐D08 treatment were determined after EAE induction. n = 11 mice for WT group and n = 9 mice for Kir4.1 cKO group, two‐tailed unpaired t‐test, **p < 0.01. L) Grid walking task showed a failed recovery of footfalls in Kir4.1 cKO mice compared with that in WT mice when both were treated with 2‐D08 after EAE. n = 11 mice for the WT group and n = 9 mice for the Kir4.1 cKO group, two‐tailed unpaired t‐test, *p < 0.05.

To directly evaluate the impact of 2‐D08 treatment on brain functional recovery in EAE mice, we conducted a series of behavioral tests following MOG_35–55_‐induced demyelination. First, we examined motor neuron function in EAE mice following the administration of 2‐D08. Compared to the vehicle group, both the number of motor neurons (**Figure** **4**A–D) and the amplitude of motor evoked potentials (MEPs) (Figure 4E–G) were significantly increased. Moreover, the beneficial effects of 2‐D08 on motor neuron recovery and MEPs were disrupted when Kir4.1 was specifically knocked out in OPCs after the onset of EAE (Figure S15A, B, Supporting Information). Second, we investigated the effect of 2‐D08 on sensorimotor functions, specifically in grid walking and inclined plane tests. The inclined plane test results showed continuous improvement in the motor abilities of EAE mice treated with 2‐D08 when placed on an inclined plane (Figure 4H). Additionally, the administration of 2‐D08 in EAE mice resulted in improved hind limb placement during free walking on the grid, leading to a significant reduction in paw‐slips compared to the vehicle group (Figure 4I). However, these improvements in movement capability were completely dissipated in Kir4.1 cKO mice in the presence of 2‐D08 after EAE (Figure 4J–L). Therefore, these findings suggest that the protective effects of 2‐D08 administration on neuronal functional recovery and movement capability primarily stem from the activation of Kir4.1 channels in vivo.

### 2.5 2‐D08 Promotes OPCs Differentiation via FYN Signaling Pathway Phosphorylation

**Figure 5:**
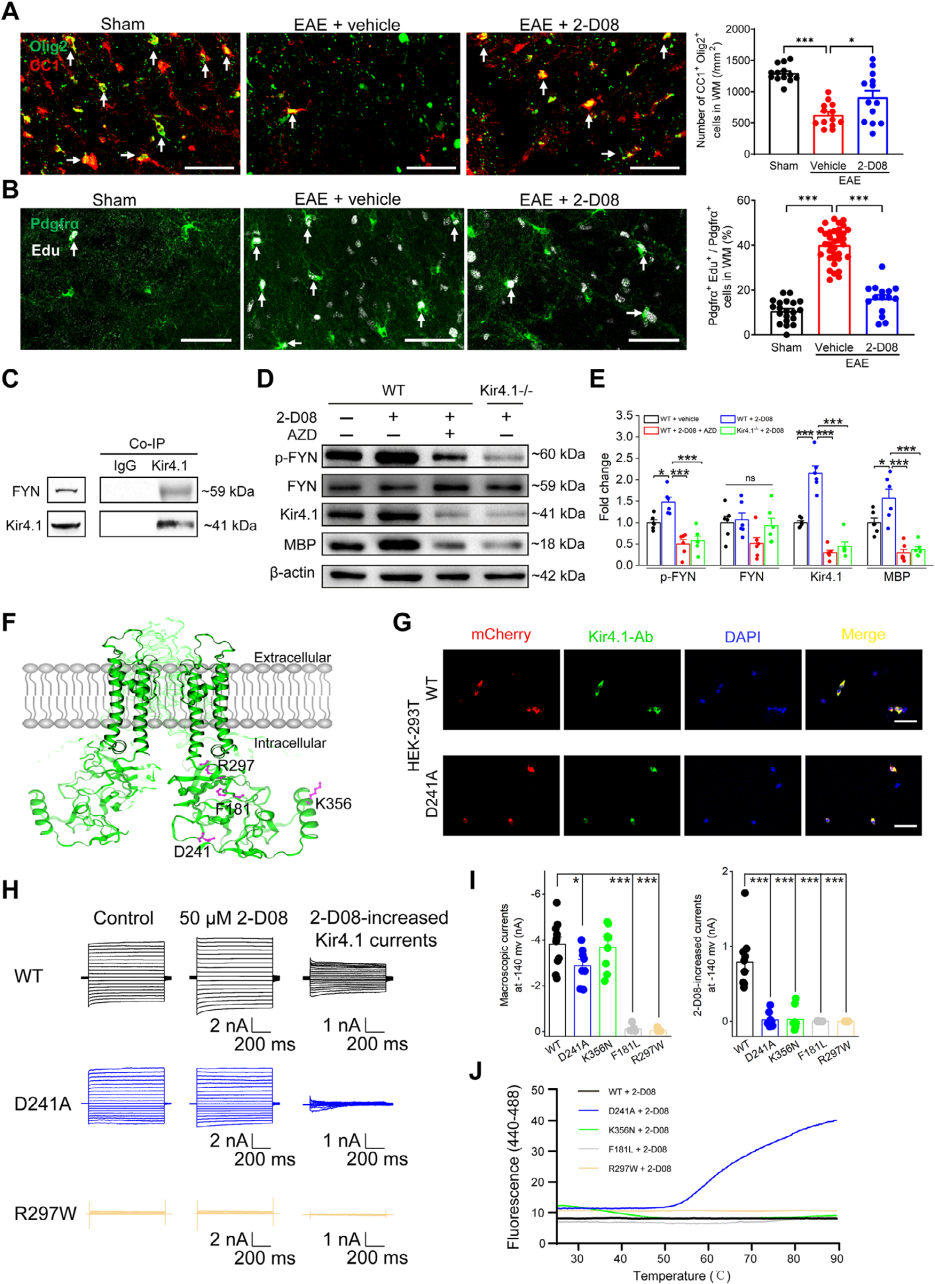
2‐D08 promoted OPCs differentiation through the phosphorylation of FYN signaling pathway. A,B) Representative immunohistochemical images and bar graphs illustrated the CC1/Olig2 co‐labeled mature oligodendrocytes (as indicated with white arrows) (A) and Edu labeled proliferating OPCs (as indicated with white arrows) (B) in the ventral horn of the spinal cord. For CC1/Olig2 co‐labeled mature oligodendrocytes analysis, n = 3 mice for each group, One‐way ANOVA with Kruskal–Wallis multiple comparisons test, *p < 0.05, ***p < 0.001. For Edu labeled proliferating OPCs analysis, n = 3 mice for each group, one‐way ANOVA with Kruskal–Wallis multiple comparisons test, ***p < 0.001. Scale bars: 50 µm. C) The co‐immunoprecipitation graph showed FYN protein reciprocally associated with Kir4.1 in OPCs. D,E) Representative western blot images (D) and the summary graph (E) of the protein levels of p‐FYN, FYN, Kir4.1 and MBP in primary cultured OPCs in the presence or absence of 2‐D08 (50 µm) and AZD0530 (2 µm) treated for 72 h (n = 6 repeated experiments). One‐way ANOVA with Tukey–Kramer multiple comparisons test, *p < 0.05, ***p < 0.001, ns: not significant. F) The simulated structure of Kir4.1 channel and its potential binding site with 2‐D08. F181, D241, R297 were predicted by AutoDock 4.0 software and K356 was predicted from the GPS‐SUMO database. G) Representative immunostaining images illustrated that the mutant D241A plasmid (red) transfected in HEK‐293T cells was colocalized with Kir4.1‐Antibody (green), nuclei were labeled blue with DAPI. Scale bars, 50 µm. H,I) Representative traces (H) and bar graphs (I) showed 2‐D08‐induced macroscopic currents and Ba^2+^‐sensitive Kir4.1 currents in HEK‐293T cells transfected with Kir4.1‐WT (n = 10 cells) or D241A (n = 8 cells), K356N (n = 10 cells), F181L (n = 6 cells), R297W (n = 7 cells) mutant plasmid, two‐tailed unpaired t‐test between each mutant plasmid and WT, *p < 0.05, ***p < 0.001. J) Typical melting curves were obtained from four mutants of the Kir4.1 channel by monitoring the thermal denaturation of the Kir4.1 protein with increasing temperature. Notably, among the four Kir4.1 mutants tested, only the D214A mutation completely abolished 2‐D08's binding affinity to Kir4.1.

In the CNS, matured oligodendrocytes are crucial for myelin formation and remyelination in both physiological and pathological conditions.^[^ ^40^ ^]^ To further explore the underlying mechanism of how oligodendrocyte lineage cells respond to 2‐D08 treatment in EAE mice, we initially analyzed the CC1^+^/Olig2^+^ immuno‐labeled mature oligodendrocytes and Edu‐labeled proliferating OPCs. We found a significant increase in matured oligodendrocytes and a decrease in the proliferation of OPCs in demyelinated spinal cord of EAE mice following 2‐D08 administration compared to untreated EAE mice (**Figure** **5**A,B). Although there was a slight increase in Edu^+^ OPCs in 2‐D08 + EAE group, this change was not statistically significant compared to sham mice. In contrast, the increase in CC1‐immunolabeled oligodendrocytes was abrogated when Kir4.1 was knocked out in OPCs following 2‐D08 administration in EAE mice (Figure S15C, Supporting Information). These results indicate that Kir4.1 channel activation by 2‐D08 in vivo accelerates the differentiation of OPCs into mature oligodendrocytes in demyelinated EAE mice.

Our previous study has demonstrated that myelin‐associated glycoprotein (MAG) interacts with FYN tyrosine kinase, leading to increased phosphorylation of FYN and activation of FYN/MYRF signals, which promote myelin formation and remyelination.^[^ ^30^ ^]^ Given this, we wondered whether Kir4.1 channel activation affects FYN signal transduction in OPCs. Indeed, co‐immunoprecipitation analysis revealed that FYN protein can associate with Kir4.1 protein, suggesting that Kir4.1 acts as an upstream pathway to activate FYN/MYRF signaling and exert myelin repair in 2‐D08 treatment (Figure 5C). Interestingly, when we incubated 2‐D08 in primary cultured OPCs for 72 h, we observed enhanced expressions of both Kir4.1 and MBP protein, along with a significant increase in the phosphorylation level of FYN, without altering the total protein expression of FYN (Figure 5D,E). In contrast, the specific FYN inhibitor, AZD0530, completely abolished the aforementioned increases in Kir4.1, MBP, and p‐FYN expressions at the same treatment duration (Figure 5D,E). Furthermore, in primary cultured OPCs obtained from Pdgfrα‐creER^TM^; Kir4.1^f/f^ transgenic mice, the enhanced phosphorylation of FYN, Kir4.1, and MBP expressions induced by 2‐D08 treatment were all dissipated, mirroring the effect of AZD0530 (Figure 5D,E). Given that 2‐D08 is widely utilized as a SUMOylation inhibitor, we next investigated whether the SUMO pathway regulates OPC differentiation. Primary OPCs cultures were treated with either a SUMO activator (N106) or inhibitor (TAK981) under standard differentiation conditions. Notably, neither SUMO pathway activation nor inhibition altered OPCs differentiation capacity (Figure S16, Supporting Information). Taken together, these results indicate that Kir4.1/FYN signaling, rather than SUMO pathway modulation, mediates 2‐D08's promotion of OPCs differentiation and myelin repair in EAE mice.

Building on our functional characterization of 2‐D08's effects on Kir4.1 activity (Figures 3, 4, 5A–E), we next sought to elucidate the structural basis of this interaction. We utilized a recently reported crystal structure of Kir4.1 and employed molecular docking software to predict potential binding sites for 2‐D08 within the Kir4.1 protein.^[^ ^41^ ^]^ As depicted in Figure 5F, the Kir4.1 structural model supports the critical involvement of residues F181, D241, R297, and K356 for the 2‐D08 binding sites, as analyzed by Autodock 4.0 software. To validate these potential residues, we introduced mutations in the aforementioned amino acids within *kcnj10*, resulting in mutants *kcnj10*‐F181L, D241A, R297W, and K356N, respectively. After transfecting the four mutant plasmids into HEK‐293T cells (Figure 5G), electrophysiological recordings revealed that when the cells carried the K356N mutation, it did not change the total K^+^ current compared to the WT. Additionally, cells carrying the F181L and R297W mutants essentially blocked total Kir4.1 currents (Figure 5I, left panel). Interestingly, the D241A mutant partially suppressed total Kir4.1 currents, demonstrating an ≈20% reduction. This aligns with the alterations observed in Kir4.1 channel currents during the EAE process. However, 2‐D08 failed to augment channel activity compared to WT plasmid transfection (Figure 5H,I). Furthermore, we conducted the TSA assay to test the binding properties of 2‐D08 with various Kir4.1 mutants. Among the four Kir4.1 mutants tested (D214A, K356N, F181L, and R297W), only the D214A mutation completely abolished 2‐D08's binding affinity to Kir4.1. In contrast, the other three mutants maintained binding profiles comparable to those of the WT Kir4.1 protein (Figure 5J). Taken together, these results suggest that D241 could be a potential binding site for 2‐D08 to activate Kir4.1 channels in the context of EAE.

### 2.6 Preclinical Assessment of 2‐D08 on Myelin Repair and Locomotor Functional Recovery in both EAE Mice and Primates

**Figure 6:**
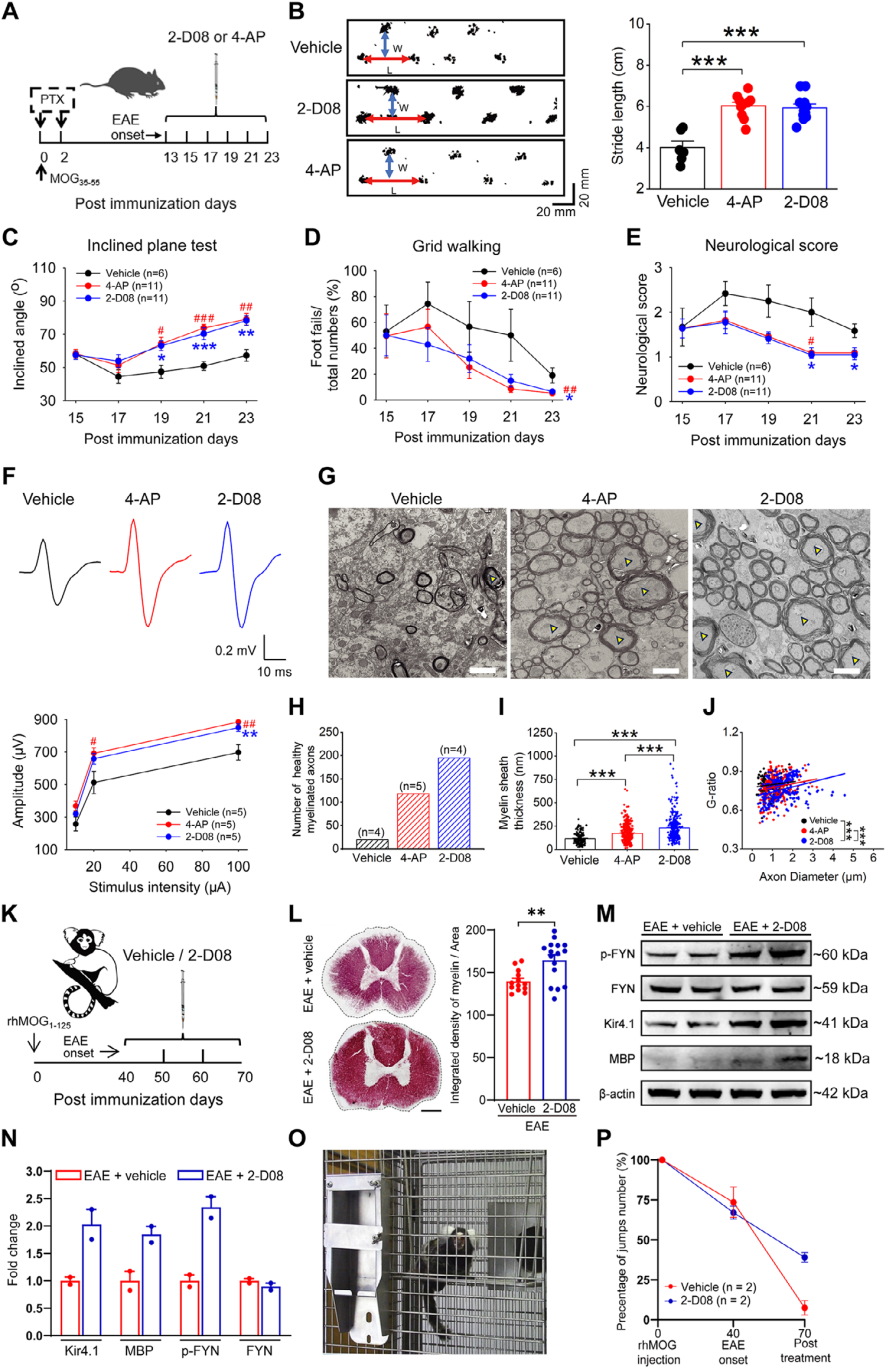
Preclinical assessment of 2‐D08 on myelin repair and locomotor functional recovery in both EAE mice and marmosets. A) The cartoon illustrated the experimental diagram in which EAE mice were treated either with 2‐D08 or 4‐AP (i.p.) starting on the disease onset. B) Representative images and the bar graph summary of the footprint tracks in EAE mice treated with 4‐AP or 2‐D08. n = 6 mice for the vehicle group and n = 11 mice for each of the 2‐D08 and 4‐AP‐treated groups, one‐way ANOVA with Tukey‐Kramer multiple comparisons test, ***p < 0.001. C–E) Inclined angle (C), grid walking task (D) and neurological scores (E) were evaluated after EAE induction with 2‐D08 or 4‐AP treatment. n = 6 mice for the vehicle group and n = 11 mice for each of the 2‐D08 and 4‐AP‐treated groups. For inclined angle after EAE analysis, one‐way ANOVA with Tukey–Kramer multiple comparisons test. For percentage of foot fails after EAE analysis, one‐way ANOVA with Kruskal‐Wallis multiple comparisons test. For neurological score analysis, one‐way ANOVA with Kruskal–Wallis multiple comparisons test. * represented the comparison between the vehicle and 2‐D08 treated mice, ^#^ represented the comparison between the vehicle and 4‐AP treated mice. F) Representative recordings of MEPs from vehicle, 4‐AP and 2‐D08‐treated mice (upper panel) and the summary bar graph of MEP amplitudes (lower panel) in each condition, n = 5 mice for each group. For MEP amplitude at stimulus intensity of 20 µA analysis, one‐way ANOVA with Kruskal–Wallis multiple comparisons test. For MEP amplitude at stimulus intensity of 100 µA analysis, one‐way ANOVA with Tukey–Kramer multiple comparisons test. * represented the comparison between the vehicle and 2‐D08 treated mice, ^#^ represented the comparison between the vehicle and 4‐AP treated mice. G–J) Electron microscopy analyzed axons from vehicle, 4‐AP and 2‐D08‐treated EAE mice. Representative electron micrographs of myelinated axons (the yellow triangles mark healthy myelin sheaths) (G), the number of healthy myelinated axons (myelin sheaths thickness > 200 nm) (H), myelin sheath thickness (I) and G‐ratio (J). Sale bars, 2 µm. n = 4 mice for vehicle, n = 5 mice for the 4‐AP‐treated group, and n = 4 mice for the 2‐D08‐treated group. For myelin sheath thickness analysis, ANOVA with Kruskal‐Wallis multiple comparisons test, ***p < 0.001. For G‐ratio of myelinated axons analysis, simple linear regression of intercepts, ***p < 0.001, Kruskal–Wallis with Dunn's multiple comparisons test. K) The cartoon illustrated the experimental scheme in which EAE marmosets were treated with either 2‐D08 or vehicle starting on day 40 after the disease onset. L) Representative gold myelin staining images and a bar graph summarizing the integrated density of myelin in the white matter of the ventral spinal cord of both EAE + vehicle and EAE + 2‐D08 treated marmosets. The data points were taken from different spinal cord sections in each marmoset (n = 2 marmosets per group). Scale bar, 20 µm. M,N) Representative images (M) and summary graph (N) showed the protein levels of p‐FYN, FYN, Kir4.1 and MBP in EAE marmosets treated with or without 2‐D08 (n = 2 marmosets for each group). O,P) The image indicated the marmoset's feeding cage, where the number of marmoset jumps was monitored (O). Bar graph (P) summarized an improved motor coordination of marmosets following 2‐D08 treatment.

We finally investigated the translational potential of 2‐D08 as a therapeutic intervention for demyelinating disorders through two assessments. Fampridine is an oral prolonged‐release formulation of 4‐AP that has been approved for the symptomatic treatment of walking disability in MS and is currently considered the most effective treatment for improving motor skills, walking ability, and walking speed.^[^ ^42^, ^43^ ^]^ In the first assessment, we compared the neuroprotective effects of 2‐D08 with 4‐AP in EAE mice. Animals were intraperitoneally injected with either 4‐AP or 2‐D08 at a dose of 1 mg kg^−1^ for 11 consecutive days, starting at the disease onset of EAE (**Figure** **6**A). Paw print analysis revealed a significant increase in stride length in both 2‐D08 and 4‐AP treated groups compared to the vehicle group (Figure 6B). Notably, both 4‐AP and 2‐D08 treatments had similar effects in ameliorating disease severity and yielded comparable results in alleviating walking disability, including the amplitudes of MEPs in vivo (Figure 6C–F). Furthermore, as 2‐D08 promotes brain functional recovery due to the axonal myelin repair in EAE lesions, we compared the effectiveness of axonal myelin repair treated by 2‐D08 or 4‐AP. Although the number of healthy myelinated axons increased in both the 2‐D08 and the 4‐AP‐treated groups (Figure 6G,H), EAE mice treated with 2‐D08 exhibited greater myelin repair of axons, as evidenced by the promoted myelin sheath thickness and the G‐ratio compared to 4‐AP‐treated EAE mice (Figure 6I,J). It is worth noting that previous studies have reported that 4‐AP has a potential risk of inducing recurrent seizures.^[^ ^44^ ^]^ In light of this, we conducted in vivo EEG recordings, and the results illustrated that, unlike 4‐AP, 2‐D08 promoted brain functional recovery without inducing seizure‐like epileptic activity in EAE mice (Figure S17A, Supporting Information).

Second, we evaluated the efficacy of 2‐D08 in marmosets utilizing the rhMOG_1‐125_ immunized EAE model, which is a valid disease model for human MS at both clinical and pathological levels.^[^ ^45^ ^]^ 2‐D08 was administered daily into the bloodstream (i.v., 1 mg kg^−1^) of marmosets at the onset of EAE symptoms, occurring 40 days post immunization (Figure 6K).^[^ ^46^ ^]^ After one month of continuous administration of 2‐D08, gold myelin staining, as well as MBP and SMI32 immunohistochemistry, revealed significant myelin repair in the spinal cords of EAE marmosets treated with 2‐D08 (Figure 6L; Figure S18A, Supporting Information). Additionally, the number of CC1^+^/Olig2^+^ labeled mature oligodendrocytes was increased compared to that in the two untreated marmosets (Figure S18B, Supporting Information). Furthermore, western blot analysis revealed an apparent increase in MBP, Kir4.1 and p‐FYN protein levels in the spinal cord after 2‐D08 treatment in EAE marmosets (Figure 6M,N), consistent with similar results found in the mice (Figure 5D,E). Finally, the 2‐D08‐treated marmosets also demonstrated a corresponding recovery of motor coordination as evidenced by the percentage of total jumps made by marmosets in the feeding cage (Figure 6O,P; Movies S1–S3, Supporting Information). In contrast, untreated marmosets progressively developed a clinical score of 3 for EAE with almost paralyzed hind legs. Collectively, these results indicate significant potential for future clinical application of 2‐D08 in MS treatment.

## 3 Discussion

The present study indicates that deficits in Kir4.1 channel function contribute to myelin loss in EAE mammals. Through TSA screening, we identified a small molecule compound, 2‐D08, as an effective activator of the Kir4.1 channel. Treatment with 2‐D08 directly promotes axonal myelin repair and improves walking and motor functions in EAE mammals, without inducing seizure‐like epileptic activity or causing obvious peripheral/cardiac toxicity (Figures S17 and S19, Supporting Information). This neuroprotective effect was mainly mediated through enhanced phosphorylation of FYN tyrosine kinase, induced by the Kir4.1 channel activator 2‐D08. Subsequently, this activation triggers the FYN/MYRF/MBP signaling pathway, promoting the differentiation of OPCs into mature oligodendrocytes.^[^ ^14^, ^47^, ^48^, ^49^, ^50^ ^]^ Our findings highlight the potential of targeting the Kir4.1 ion channel in OPCs as an approach for protecting neurons from demyelinating injuries. It suggests that 2‐D08 treatment holds promise as a therapeutic strategy for demyelinating diseases, including MS.

The Kir4.1 channel is considered a neurophysiological marker of OPCs and is widely expressed in astrocyte and oligodendrocyte lineage cells. It plays a vital role in regulating the resting membrane potential of these cells and facilitates the maturation of oligodendrocytes. In white matter, Kir4.1 is crucial for maintaining extracellular potassium (K^+^) balance and axonal electrical conduction.^[^ ^13^ ^]^ Inhibition of the Kir4.1 channel impedes OPCs differentiation and oligodendrocyte maturation, potentially by affecting intracellular pH through Na^+^/H^+^ exchangers during the brain development.^[^ ^51^ ^]^ More importantly, the impairment of Kir4.1 channels in OPCs directly leads to delayed recovery of axons from repetitive stimulation in white matter, as well as spontaneous seizures.^[^ ^52^ ^]^ However, to date, there is unclear whether the *kcnj10* gene encodes a protein involved in myelin synthesis/degradation or the immune response to myelin.^[^ ^53^ ^]^

The compound 2‐D08, known as a SUMO E2 inhibitor, has been used to inhibit SUMOylation and has demonstrated various biological properties in vitro.^[^ ^54^ ^]^ A previous study showed that 2‐D08 may have a novel anti‐aggregatory and neuroprotective effect against the neurotoxicity of amyloid beta proteins in Alzheimer's disease.^[^ ^55^ ^]^ Choi et al. disclosed that 2‐D08 inhibited cell migration and invasion by mediating K‐Ras deSUMOylation in pancreatic cancer cells.^[^ ^56^ ^]^ It has also been reported that 2‐D08 induced apoptosis in a human AML cell line through the accumulation of reactive oxygen species (ROS), with Nox2 deSUMOylation potentially playing a significant role.^[^ ^57^ ^]^ These findings have generated considerable interest in the potential neuroprotective effects of 2‐D08, particularly regarding its antitumor effects.^[^ ^56^ ^]^ In the current study, we provide evidence that 2‐D08 treatment accelerates OPCs differentiation and axonal myelin repair under both physiological and pathological conditions by phosphorylating the FYN pathway through Kir4.1 channels activation at the D241 site (Figure 5C–J), suggesting a new role for 2‐D08 in the brain beyond its known SUMOylation effect. Although it is intriguing that 2‐D08 exhibits selective affinity for Kir4.1 channels expressed in OPCs over astrocytes, we speculate that this binding selectivity may stem from several key differences in Kir4.1 channel biology and cellular context. First, Kir4.1 in OPCs primarily forms homotetramers, whereas in astrocytes, it predominantly exists as heterotetramers with Kir5.1.^[^ ^58^ ^]^ This difference may create distinct binding pockets that favor 2‐D08 interaction with OPC‐specific Kir4.1 channels. Second, OPCs exhibit a more hyperpolarized resting membrane potential (−80.5 ± 0.7 mV, Figure 1G) compared to astrocytes (−76.8 ± 1.5 mV, Figure S4B, Supporting Information) in the spinal cord. This hyperpolarized membrane state in OPCs may facilitate the activation of Kir4.1 channels by 2‐D08, as the Kir4.1 channel is more likely to be in an activation‐permissive conformation under hyperpolarized conditions. Third, OPCs possess unique microenvironmental sensitivity. Studies have shown that proliferating OPCs accumulate in the lesion areas of MS patients.^[^ ^38^ ^]^ Unlike astrocytes, OPCs are the only type of glial cells that exhibit precursor cell characteristics, allowing them to dynamically adapt to environmental changes. OPCs express distinct membrane components, such as lipid rafts, developmental markers, and adhesion proteins, which could allosterically modulate Kir4.1 or enhance 2‐D08 binding. Collectively, these structural, biophysical, and microenvironmental factors may underlie the selective action of 2‐D08 on OPCs.

Mechanistically, we unexpectedly found that the membrane channel protein Kir4.1 can interact with FYN tyrosine kinase. FYN is a non‐receptor, cytoplasmic tyrosine kinase (TK) belonging to the Src family kinases (SFKs), which are involved in multiple signaling pathways in the central nervous system (CNS), including synaptic transmission, myelination, axon guidance, and oligodendrocyte formation.^[^ ^59^ ^]^ Previous studies have indicated that the downregulation of the *kcnj10* gene occurs in mice with a deletion of the myelin‐related gene *ANGPTL2*. Deletion of *ANGPTL2* can cause abnormalities in phosphorylation within the FYN pathway, which subsequently hampers oligodendrocyte differentiation and myelin regeneration.^[^ ^30^ ^]^ In fact, deletion of *ANGPTL2* causes numerous gene downregulations including *kcnj10*.*^[^* ^30^ ^]^ Building upon these findings, our investigation focused on the Kir4.1/FYN/myelin signaling pathway. Indeed, activation of Kir4.1 channels by 2‐D08 increases the phosphorylation of FYN, as well as the expression of MBP (Figure 5C–E). In contrast, Kir4.1/FYN/MBP signals were completely blocked in Kir4.1 deficient mice (Figure 5D,E), uncovering a newly identified signaling pathway of *kcnj10*‐regulated myelination. Therefore, in our current study, we propose a dual‐axis regulatory mechanism for 2‐D08. Direct binding of 2‐D08 acutely enhances Kir4.1 channel activity (Figures 2F–I, 3A–D), while subsequent Fyn recruitment promotes Kir4.1 expression and stabilization (Figure 5C–E). This dual action creates a positive feedback loop: initial activation of Kir4.1 by 2‐D08 facilitates Fyn‐mediated phosphorylation, which in turn reinforces Kir4.1 membrane stability and function. Ultimately, this process drives OPCs differentiation. Although further studies are needed to dissect the precise temporal dynamics, this synergistic mechanism explains the potent pro‐myelination effects of 2‐D08.

It is worth of mentioning that increasing evidence has demonstrated the potential of targeting ion channels to prevent neuronal damage in demyelinating diseases. Kapell et al. recently reported that neuron‐oligodendrocyte potassium shuttling at nodes of Ranvier protects against inflammatory demyelination.^[^ ^29^ ^]^ They utilized the small molecule retigabine to specifically activate Kv7, reducing the hyperexcitability of neurons and effectively treating EAE. In a parallel trial, they compared the efficacy of retigabine with 4‐AP, a drug already used clinically as an adjuvant therapy for MS, and found that retigabine outperformed 4‐AP. However, retigabine was withdrawn from the market in 2017 due to severe adverse reactions observed during its clinical use.^[^ ^60^ ^]^ Therefore, seeking safety and effective drugs has always been the focus in the MS therapy. Our data revealed that treatment with 4‐AP or 2‐D08 yielded similar results in alleviating walking disability and improving motor functions, although 2‐D08 shared a distinct cellular mechanism by targeting Kir4.1 channels and activating FYN/MYRF signals in OPCs. Previous studies have reported that 4‐AP has a potential risk of conducting recurrent seizures.^[^ ^44^ ^]^ We found increased spontaneous epileptiform events in L5 neocortical pyramidal cells during 4‐AP applications (50 µm) using whole‐cell patch recordings, while there were no obvious changes in spontaneous epileptiform events nor in RMPs of these pyramidal neurons during 2‐D08 applications (50 µm, Figure S17B, Supporting Information). Additionally, EEG recordings in vivo after 4‐AP or 2‐D08 injections showed that 4‐AP injected mice exhibited strong focal seizures starting on day 3 and showed continuous aggravation by day 5. In contrast, there were no paroxysmal seizures observed in 2‐D08 injected mice (Figure S17A, Supporting Information).

In the present study, we reported that treatment with 2‐D08, by targeting Kir4.1 channels, significantly ameliorated demyelination injuries in the CNS and correspondingly mitigated motor behavioral deficits in EAE mice and marmosets. Given the complexity of the pathogenesis of MS, it is important to acknowledge that several questions remain unanswered in our research. First, the Kir4.1 channel, as a subtype of inwardly rectifying K^+^ channels, is broadly expressed in astrocyte and oligodendrocyte lineage cells within the CNS. In the EAE mouse model of MS, we found that impairment of Kir4.1 channel activity initially occurred in OPCs (Figure 1). In line with this, we analyzed the gene bank (GSE180759) uploaded from a previous human MS study,^[^ ^61^ ^]^ which showed that the expression of the *kcnj10* gene was significantly decreased in OPCs of MS patients compared to samples from healthy controls (Mann–Whitney test, p = 0.0357). However, these MS samples were obtained from the human brain tissues, and specific brain regions, such as the spinal cord, still need to be further investigated in future human MS studies. Second, the small marmoset cohort (n = 2) in our study limits statistical power, though the consistent therapeutic effects observed provide preliminary evidence supporting 2‐D08's translational potential, further investigations in the marmoset model are warranted to validate and expand upon these preliminary findings. Third, previous studies have reported that the Kir4.1 channel expressed in mature oligodendrocytes is essential for axon health and is closely associated with myelin repair.^[^ ^29^, ^36^ ^]^ For instance, potassium shuttling at the nodes of Ranvier between neuronal Kv channels and oligodendroglial Kir4.1 channels contributes to inflammatory demyelination, and mature oligodendrocytes facilitate axonal glucose metabolism. Moreover, considering the immune response of microglia in the progression of MS,^[^ ^61^ ^]^ the potential of 2‐D08 to improve the overall niche environment of brain health during the MS process remains to be further explored.

## 4 Experimental Section

### Antibodies and Reagents

All commercial antibodies, reagents, and kits utilized in this study are detailed in Table S2 (Supporting Information).

### Animals

Pdgfrα‐creER^TM^ (JAX strain 018280), Kir4.1^f/f^ (JAX strain 026826), and Rosa26‐mGFP (JAX strain 007676) were obtained from the Jackson Laboratory (USA). C57BL/6J mice (JAX strain 000664) were obtained from the Slac Laboratory Animal (Shanghai, China). Cre recombinase induction in Pdgfrα‐creER^TM^; Kir4.1^f/f^ mice and Pdgfrα‐creER^TM^; mGFP mice were achieved via intraperitoneal injection of tamoxifen (120 mg kg^−1^ in corn oil) for five consecutive days. The animal experiment protocols were authorized by the US National Institutes of Health (Protocol number: A‐2022‐036) and were approved by the Animal Ethics Committee of Shanghai Jiao Tong University School of Medicine (AAALAC accreditation Unit, 001670).

All healthy marmosets (Callithrix jacchus) used in this study were housed at the Institute of Neuroscience, Center for Excellence in Brain Science and Intelligence Technology, Chinese Academy of Sciences, in accordance with the standards of the Institute of Animal Care Committee of the Center for Excellence in Brain Science and Intelligence Technology, Chinese Academy of Sciences (No. CEBSIT‐2023020). Two male and two female adult marmosets were included in this study.

### Human Serum Samples

Serum samples from MS patients and healthy controls were obtained from Renji Hospital, Shanghai Jiao Tong University School of Medicine and received ethical approval from the human ethical committee in Renji Hospital, Shanghai Jiao Tong University School of Medicine, China (Reference No. KY‐2021‐137‐B). Written informed consent was obtained from all participants.

### EAE Models in Mice and Marmosets

Mouse EAE induction was performed as previously reported.^[^ ^62^ ^]^ Briefly, adult male C57BL/6J mice were immunized subcutaneously with MOG_35‐55_ (300 µg/mouse) emulsified in complete Freund's adjuvant (CFA) containing mycobacterium tuberculosis (Sigma, F5881) according to previous study. Pertussis toxin (200 ng/mouse) in saline was administered intraperitoneally on days 0 and 2. Clinical scores were assessed daily by blinded researchers using a 0–5 scale: 0 (no symptoms), 1 (tail paralysis), 2 (paresis), 3 (paraplegia), 4 (forelimb weakness), and 5 (moribund/death).^[^ ^63^ ^]^

Marmoset EAE model was performed as previously reported.^[^ ^64^, ^65^ ^]^ Briefly, 100 µg rhMOG_1‐125_ in 300 µL PBS and was emulsified in 300 µL CFA containing mycobacterium butyricum (Sigma, F5881) by gentle stirring for at least 1 h in ice. Under isoflurane anesthesia, all marmosets were injected with 600 µL of emulsion into the dorsal skin at four sites, two in the inguinal region and two in the axillary region. Pertussis toxin was not used in the induction of EAE in marmosets. Neurological symptoms were scored as previously described semiquantitative scale^[^ ^64^ ^]^: 0 (asymptomatic), 0.5 (mild behavioral changes), 1 (lethargy), 2 (ataxia), 2.5 (paresis/brainstem involvement), 3 (paraplegia), 4 (quadriplegia), and 5 (death). Animals reaching a score of 3 were euthanized.

### LPC‐Induced Focal Demyelination Model in Mouse Spinal Cord

Adult C57BL/6 mice were anesthetized with 1% pentobarbital and securely positioned in a stereotaxic apparatus (RWD Instruments, Shenzhen, China). The core body temperature was maintained at 37 °C using a feedback‐controlled heating pad throughout the surgical procedure. After laminectomy at the thoracic level (T8‐T10), the dura mater was carefully removed. Focal demyelination was induced by unilateral injection of 1% lysolecithin dissolved in sterile PBS. Using a glass micropipette (tip diameter 20–30 µm) connected to a microinjection system (Nanoject III, Drummond), 1 µL of LPC solution was slowly delivered into the ventral spinal cord at a rate of 100 nL min^−1^. The pipette was left in place for an additional 5 min post‐injection to prevent backflow. After surgery, the muscle layers and the skin were sutured.

### Construction of pLVX‐Kcnj10‐IRES‐mCherry Plasmid

The pLVX‐Kcnj10‐IRES‐mCherry plasmid was generated by cloning Kcnj10 cDNA (NM_0 02241.5) into pLVX‐IRES‐mCherry via Xbal / BamHI restriction sites to obtain all functional domains required for proper channel activity. As *Kcnj10* gene is highly conserved across mammalian species, with >95% amino acid identity between human and rodent orthologs, particularly in the pore‐forming regions and key functional domains. A conserved domain primers was used:

forward (XbaI): 5′GCTCTAGAATGACGTCAGTTGCCAAGGTG3',
reverse (BamHI): 5′CGGGATCCTCAGACATTGCTGATGCGCAC3' (Sangon Biotech, Shanghai, China).

### Thermal Shift Assay

The protein thermal shift assay was performed as previously reported.^[^ ^66^ ^]^ The protein solution was mixed with the fluorescent probe SYPRO‐orange, followed by gradual heating from 25 to 99 °C). The Kir4.1 protein was diluted to a concentration of 20 µL in a reaction buffer comprising 1 µL of the testing compounds, 10X SYPRO‐orange and phosphate buffer. The protocols in the thermo Lightcycler 480 were: 25 °C for 2 min, then to 95 °C, with temperature increments of 0.3 °C every 15 s. Fluorescence intensity was measured at each temperature. Each condition was duplicated twice.

### Cell Culture and Transfection

HEK‐293T cells (ATCC CRL‐1573) were maintained in DMEM supplemented with 10% FBS and 1% penicillin / streptomycin. Cells were transiently transfected with *Kcnj10* plasmids using Lipofectamine 2000 (Invitrogen) according to the standard protocol. For patch‐clamp recordings, Kir4.1‐expressing cells were trypsinized and seeded onto 12‐mm coverslips on the day of experimentation.

As for the primary OPCs culture,^[^ ^35^ ^]^ cortical tissues from embryos or postnatal day 1 mice were dissected, minced, and digested in trypsin/DNase I solution. After centrifugation, cells were resuspended in neurosphere growth medium (DMEM/F12 with B27, EGF and bFGF) and cultured for 8–10 days. Neurospheres were then transferred to oligosphere medium (DMEM/F12 containing PDGF, bFGF) for 7–9 days, oligospheres were plated on a PDL‐coated dish in OPCs medium (DMEM/F12 supplemented with B27, N2, 0.1% BSA, 10 ng mL^−1^ PDGF, 20 ng mL^−1^ bFGF, 5 µg mL^−1^ IGF).

To obtain mature oligodendrocytes, purified OPCs were cultured in neurobasal/B27 medium for 3 days to facilitate differentiation.

### Astrocytes and OPCs Isolation by FACS

For cell‐type specific isolation, astrocytes and OPCs were fluorescently labeled in vivo prior to sorting. Astrocytes were tagged in C57BL/6J mice through orbital injection of rAAV2/PHP.eB‐GFAP‐EGFP virus, while OPCs were genetically labeled in Pdgfrα‐creER^TM^; mGFP transgenic mice. Following a 21‐days expression period, anesthetized mice were perfused and neural tissues (brain and spinal cord) were collected for processing. Tissue dissociation was performed using an optimized enzymatic protocol. FACS was conducted using a high‐speed cell sorter (BD FACS Aria II Flow Cytomerter) with a 70 µm nozzle, applying stringent gating parameters to ensure population purity. For the extraction of the Kir4.1 protein, cells were processed for protein extraction using ice‐cold RIPA buffer containing protease inhibitors. Kir4.1 channel proteins were immunoprecipitated with subtype‐specific antibodies coupled to magnetic beads, followed by elution in phosphate buffer for subsequent thermal shift assays.

### Single‐Cell RT‐PCR

For single‐cell gene expression analysis, microaspiration technique was employed to isolate GFP‐positive OPCs from Pdgfrα‐creER^TM^; mGFP and Pdgfrα‐creER^TM^; mGFP; Kir4.1^f/f^ mice as previously described.^[^ ^49^ ^]^ Briefly, micromanipulation under fluorescence guidance, individual GFP^+^ cells were captured and rapidly transferred to chilled lysis buffer. To preserve native transcriptional profiles, the entire collection process was completed within a 180‐min window following tissue sectioning, with subsequent RNA processing initiated within 60 min of cell isolation. For single cell transcriptome analysis, cell lysis, RNA extraction and reverse transcription to cDNA were performed. Single‐cell cDNA was amplified using KAPA HiFi HotStart ReadyMix according to the manufacturer's protocol.^[^ ^67^ ^]^ Gapdh was used as an internal control.

### Detection of Serum Anti‐Kir4.1 Antibodies by ELISA

The ELISA for detecting anti‐Kir4.1 antibodies was performed using purified human Kir4.1 protein as the capture antigen. The Kir4.1 protein was recombinantly expressed in HEK‐293T cells following transfection with a Kir4.1‐encoding plasmid, after which it was extracted and purified under native conditions to preserve conformational epitopes. The purified protein was subsequently coated onto high‐binding 96‐well microplates at an optimized concentration of 2 µg mL^−1^ in phosphate buffer and incubated overnight at 4 °C to ensure efficient adsorption. All ELISA detecting steps were performed according to the standardized protocol provided by Xiamen Lun Changshuo Biotechnology Co. Ltd. and different concentrations of positive controls were added to ensure the specificity and accuracy of the assay.

### Protein Extraction and Western Blotting

For western blotting analysis, tissue and cell samples were homogenized in ice‐cold lysis buffer and clarified by centrifugation (12 000 × g, 20 min, 4 °C). Protein concentration in the supernatant was determined by bicinchoninic acid assay before heat denaturation at 65 °C for 30 min. Proteins were resolved by SDS‐PAGE using Tris‐glycine gels and subsequently transferred to PVDF membranes. After blocking with 3% BSA in TBST (TBS with 0.1% Tween 20, pH 7.6) for 2 h at room temperature, membranes were probed with primary antibodies overnight at 4 °C. Following extensive washing, membranes were incubated with species‐appropriate HRP‐conjugated secondary antibodies (1:5000) for 2 h at room temperature. Protein bands were detected using enhanced chemiluminescence substrate and imaged with Tanon Chemiluminescence imaging system. To ensure equal loading, β‐actin expression was assessed in parallel. Densitometric analysis was performed using ImageJ software (NIH) for quantitative comparisons between samples.^[^ ^35^ ^]^

### Immunohistochemistry and Image Analysis

For immunohistochemical characterization, mice underwent transcardial perfusion with physiological saline followed by 4% paraformaldehyde in phosphate buffer. After overnight post‐fixation at 4 °C, spinal cord tissues were sectioned sagittally at 40 µm thickness using a vibratome. Free‐floating sections underwent permeabilization with Triton X‐100 and blocking with normal serum before incubation with primary antibodies targeting neural lineage markers (including NG2, Pdgfrα, GFAP, Kir4.1, CC1, GFP, mCherry, S100β, ChAT, NeuN, Olig2, SMI32 and MBP) at optimized concentrations. Following extensive washing, antigen‐antibody complexes were visualized using species‐specific secondary antibodies conjugated to Alexa Fluor dyes. Nuclear counterstaining was performed with DAPI prior to mounting in aqueous medium.

For cell proliferation studies, EdU (50 mg kg^−1^) was administered intraperitoneally for five consecutive days starting at EAE onset, subsequent labeled with the Click‐It Plus Alexa Fluor 647 Picolyl Azide Toolkit (Molecular Probes) using 200 nm dye azide. All images were acquired on a Leica TCS SP8 confocal microscope at the Core Facility of Basic Medical Sciences, Shanghai Jiao Tong University School of Medicine and analyzed using ImageJ software for threshold‐based quantification andadvanced morphometric analysis, ensuring consistent and reproducible measurements across experimental groups.

### Gold Myelin Staining

Gold myelin staining was performed according to the manufacturer's instructions. Briefly, spinal cord tissues were sectioned sagittally at 40 µm thickness using a vibratome. After bringing the sections to room temperature, they were dried at 37 °C for 30 min to enhance tissue adhesion. The staining reagent and stop buffer were freshly prepared by diluting the stock solutions with deionized water according to the recommended working concentrations. The staining solution was then applied to the sections, which were incubated in a humidified chamber at 45 °C for 20–30 min in the dark. After incubation, the sections were rinsed twice with deionized water (1 min per wash) to remove unbound dye. The reaction was terminated by applying the stop buffer and incubating at 45 °C for 2–3 min, followed by three additional washes in deionized water (1 min each).

### Transmission Electron Microscopy

For ultrastructural examination, anesthetized mice underwent transcardial perfusion with ice‐cold PBS followed by 2.5% glutaraldehyde in 0.1 m PB (pH 7.4) according to previously study.^[^ ^35^ ^]^ After overnight post‐fixation at 4 °C in the same fixative, spinal cord tissues were thoroughly rinsed and subsequently treated with 1% osmium tetroxide for 60 min at room temperature. The samples then underwent progressive dehydration through an ethanol gradient series (30–100%), during which en bloc staining was performed using 1% uranyl acetate in 70% ethanol. Following complete dehydration and propylene oxide clearing, tissues were infiltrated with epoxy resin overnight and polymerized at 60 °C for 72 h. Ultrathin sections (90 nm thick) were prepared using an ultramicrotome equipped with a diamond knife, mounted on copper grids, and examined under standard TEM operating conditions (120 kV). Digital image acquisition was performed using an integrated CCD camera system at Shanghai Jiao Tong University with particular attention paid to myelin sheath structures.

### Elemental Quantification of [K^+^]_o_

To determine in vivo potassium ion levels, sham and EAE mice were anaesthetised with 1% pentobarbital and the shielded K^+^ nanosensors (20 µg mL^−1^, 5 µL) were injected into the white matter of the ventral spinal cord (DV, −1.2 mm). A blue laser light source was delivered to the same white matter area through an optical fiber connected to the fiber optic photometry system (Inper fibre photometry system). After the mice were placed in the chamber, the optical fiber was secured to ensure that it did not move during the recording. Once the signal had stabilized, a 60 s recording was made for each mouse.^[^ ^34^ ^]^

### Mouse Spinal Cord Virus Injection

Adult mice were anesthetized with 1% pentobarbital and mounted in a stereotaxic frame (RWD Instruments, Shenzhen, China). Body temperature was maintained using a heating pad. An incision was made to expose the spine and holes were drilled for virus injection using glass pipettes. Viral (AAV2/5‐gfaABC1D‐ERT2‐Cre‐ERT2‐WPRE‐pA) injections were targeted to the white matter of the spinal cord (DV, −1.2 mm).

### Acute Slices Preparation

Mice were deeply anesthetized with 1% pentobarbital. The spinal cord was exposed by laminectomy and the lumbosacral segments were removed into oxygenated ice‐cold dissection solution containing (in mm): 92  NMDG, 2.5  KCl, 1.25  NaH_2_PO_4_, 30  NaHCO_3_, 20  HEPES, 25  glucose, 2  thiourea, 5  sodium ascorbate, 3  sodium pyruvate, 0.5  CaCl_2_, and 10  MgSO_4_·7H_2_O (The pH was titrated to 7.3–7.4 with concentrated HCl). After removed the dorsal roots, ventral roots and meninges, blocks of the lumbar spinal cord were embedded into agar and coronal slices were cut at 300 µm on a Vibratome (VT1000S, Leica Microsystems, Germany). For the preparation of brain slices, coronal sections of hippocampal slices were cut at 300 µm thickness.

The slices were stored in dissection solution for 10–15 min at 31 °C, and then slices were allowed to equilibrate for at least 1 h at 31 °C in normal ACSF containing (in mm): 125 NaCl, 2.5 KCl, 1 MgCl_2_, 2 CaCl_2_, 1.25 NaH_2_PO_4_, 25 NaHCO_3_, and 12.5 D‐glucose. All the buffers in this experiment were continuously bubbled with a mixture of 95% O_2_/5% CO_2_ gas.

### Electrophysiological Recordings for Acute Slices and Cultured Cells

Patch‐clamp recordings were performed on both acute brain slices and cultured cell preparations to investigate membrane properties and channel activities. Acute slices (300 µm thickness) were obtained from either Pdgfrα‐creER^TM^; mGFP transgenic mice for OPCs recordings, while astrocytes were identified based on their distinctive membrane characteristics.^[^ ^18^ ^]^ Following equilibration in oxygenated ACSF at 31 °C for ≥ 1 h, individual slices were transferred to a recording chamber maintained at room temperature (22–24 °C) with continuous ACSF perfusion. Cellular visualization was achieved using an upright microscope (BX51WI, Olympus) equipped with differential interference contrast optics and infrared‐sensitive camera (optiMOS, QIMAGING). Whole‐cell recordings were acquired using a Multiclamp 700B amplifier connected to a Digidata 1550A interface, with signals filtered at 2 kHz and digitized at 20 kHz sampling rate. Borosilicate glass pipettes (6–8 MΩ resistance) were fabricated using a programmable puller (Sutter P‐1000) and filled with intracellular solution containing (in mm): 125 K‐gluconate, 15 KCl, 8 NaCl, 10 HEPES, 0.2 EGTA, 3 Na_2_‐ATP, and 0.3 Na‐GTP (pH 7.3). For morphological reconstruction, 20 µm Alexa Fluor 568 was included in the pipette solution.

Parallel experiments in HEK‐293T cells involved transient transfection with Kcnj10‐IRES‐mCherry plasmid 24 h prior to recording. Fluorescently labeled cells were targeted for whole‐cell configuration using lower‐resistance pipettes (2–3 MΩ) and perfused with extracellular solution containing (in mm): 150 NaCl, 10 glucose, 10 HEPES, 2 CaCl_2_, 5 KCl, and 1 MgCl_2_ (pH 7.3). Kir4.1‐specific currents were isolated by bath application of 100 µm BaCl_2_, a selective channel blocker.

### Motor Evoked Potential (MEP) Recordings In Vivo

Mice were anesthetized with 1% pentobarbital via intraperitoneal injection and the gastrocnemius muscle were exposed. A bipolar hook stimulating electrode was placed on the motor cortex on the contralateral side. The ground electrode was placed in a superficial muscle layer around the skin. Responses to the stimulation were recorded from the gastrocnemius muscle using a NeuroLego amplifier (Jiangsu Brain Medical Technology Co.ltd, Nanjing, China) and digitized at 30 kHz. To ensure a maximum waveform and prevent independent muscle contractions, the stimulating intensity was set to 1–10 mA, the duration to 0.2 ms, and the frequency to 1 Hz. In addition, smaller stimulating intensities at 10–100 µA were also trigged using stimulus generator (Multichannel Systems STG4008, programmed with MC Stimulus II software). Responses of the gastrocnemius muscle were recorded using a 250 µm diameter 304 stainless steel electrode (Kedou Brain Computer Technology Co. Ltd., Suzhou, China). Date were acquired using an electrophysiology data acquisition system (Medusa, Bio‐Signal Technologies, USA) and digitized at 1 kHz. Signals were extracted with a 50 Hz filter and then stored for further analysis.

### EEG Recordings

Mice were anesthetized with 1% pentobarbital via intraperitoneal injection and placed into a stereotaxic apparatus for the electrode implantation. Electrodes were implanted into the left dorsal hippocampus (AP: −1.8, ML: +2.0, DV: −2.0) for EEG recording. One screw electrode was positioned in the skull and used as ground electrode (frontal location). After one week recovery from the surgery, baseline activities were recorded. After three days of recording baseline activity, mice were injected with 4‐AP or 2‐D08 (1 mg kg^−1^). The acquired EEG signals were processed through a high‐performance analog‐to‐digital conversion system (Power Lab 8/30 ML870) with subsequent analysis conducted in LabChart 8.0 software environment. Continuous recordings were obtained at 1 kHz sampling frequency with application of a 500 Hz low‐pass filter to ensure optimal signal fidelity. Epileptiform activity was identified through systematic visual inspection of the recordings, with abnormal events defined as sustained (≥10 s duration) rhythmic spike or sharp‐wave complexes exhibiting amplitude ≥200% of baseline activity, consistent with established criteria for epileptiform discharges in rodent models.

### Drug Delivery

2‐D08 (HPLC > 98%) was purchased from TargetMol (Shanghai Tao Shu Biotechnology Co., LTD). It was dissolved in dimethyl sulfoxide (DMSO) to make stock solutions and was kept at −20 °C. 4‐AP (HPLC > 99%) was purchased from Sigma–Aldrich (504‐24‐5). The stock solution was diluted with proper solution according to the manufacturer's guidelines. EAE mice were administered 1 mg kg^−1^ of 2‐D08, 4‐AP or vehicle via daily intraperitoneal (i.p.) injection, starting 48 h after immunization (prophylactic treatment) or at the onset of the disease symptom (therapeutic treatment). For EAE in marmosets, treatment with 2‐D08 or vehicle began on day 40 after immunization and continued for 30 days.

### Grid‐Walking Task

To evaluate sensorimotor coordination and locomotor function, a standardized grid‐walking paradigm adapted from established protocols was employed.^[^ ^35^ ^]^ Animals were individually positioned within an open‐field grid enclosure (35×25×40 cm) featuring 15 mm square apertures, permitting unrestricted ambulation for a 5‐min observation period. Limb placement errors were quantified according to two criteria: 1) complete limb protrusion through the grid opening, or 2) sustained limb positioning with the wrist joint at or below the grid plane. Trained observers, maintained unaware of experimental groupings, documented both successful steps and placement errors. The error frequency was derived using the equation: (error count)/ (total steps taken) × 100%, where total steps represented the sum of erroneous and proper limb placements.

### Inclined Plane Test

To evaluate sensorimotor function, mice were subjected to an inclined plane test adapted from established protocols.^[^ ^68^ ^]^ Animals were positioned horizontally on an adjustable platform with their longitudinal axis perpendicular to the inclination plane. The apparatus was gradually tilted in 5° increments until reaching the maximum angle at which each mouse could maintain position for 5 s without sliding. This critical angle was determined through three consecutive trials with appropriate rest intervals, and the mean value was calculated for statistical analysis. The standardized protocol ensured consistent assessment of vestibulomotor function while minimizing inter‐trial variability.

### Paw Print Analysis

The procedure was conducted as described before with slight modifications.^[^ ^18^ ^]^ Mice were trained to walk straight across a runway (40 cm long and 4 cm wide) three times for acclimatization. Subsequently, mice were required to traverse the straight runway with white paper to obtain an edible treat. The hind paws were dipped in nontoxic black paint. The footprints were analyzed to collect stride length and width, unclear and partial paw prints were excluded from the analysis.

### Statistical Analysis

All quantitative data analysis was performed using GraphPad InStat 3 software, with graphical representations generated through Origin 8. Experimental results are expressed as mean values accompanied by standard error of the mean (SEM). The statistical approach was guided by initial assessment of data distribution characteristics – normality testing determined the subsequent selection of appropriate analytical methods. For datasets conforming to normal distribution, parametric analyses including both paired and unpaired two‐tailed Student's t‐tests were employed, supplemented by one‐way ANOVA with Tukey–Kramer post‐hoc comparisons. Non‐normally distributed datasets were analyzed using non‐parametric alternatives: Mann–Whitney tests for independent samples, Wilcoxon tests for paired comparisons, and one‐way ANOVA with Kruskal–Wallis tests for multi‐group analyses. Sample sizes varied by experimental paradigm: electrophysiological recordings were quantified by individual cells (n), biochemical assays by animal subjects (n), and behavioral assessments by tested animals (n). All data presented in the study with mean ± s.e.m. Thresholds for statistical significance were established a priori at *p* < 0.05 (*), *p* < 0.01 (**), and *p* < 0.001 (***) levels. Throughout all experimental procedures and data analysis, investigators maintained blinding to treatment groups and sample identities.

## Conflict of Interest

The authors declare no conflict of interest.

## Author Contributions

M.L., S.J., X.F., and C.X. contributed equally to this work. X. T., N.G., and Y.G. performed conceptualization. X.T., M.L., X.F., S.J., X.H., L.C., D.S. performed methodology. X.T., M.L., X.F., S.J., X.H., X.S., L.C., Y.F., D.H., and X.Z. performed investigation. X.T., S.J., X.S., X.H., Y.F. performed data curation. M.L., X.F., S.J., and X.H. performed formal analysis. X, Tong, N.G., M.L., X.F., S.J., X.S., X.H., Y.F., and D.H. performed validation. X.T., M.L., X.F., S.J. performed visualization. X.T. performed supervision. X.T., M.L., X.F., and S.J. wrote the original draft. X.T. and X.F. reviewed and edited the final manuscript. N.G., Y.G., L.C., C.X., Y.C., D.L., F.L., Q.W., X.H., and D.H. acquired resources. X.T., L.C., and X.Z. performed project administration. X.T. performed funding acquisition. All authors approved the final version of the manuscript.

## Supporting information



Supporting Information

Supporting Information

Supporting Information

Supplemental Movie 1

Supplemental Movie 2

Supplemental Movie 3

## Data Availability

The data that support the findings of this study are available from the corresponding author upon reasonable request.
