# Supplementary material for: Activation of Kir4.1 Channels by 2‐D08 Promotes Myelin Repair in Multiple Sclerosis
Source: Adv Sci (Weinh). 2025 Jun 5;12(34):e02032. doi: 10.1002/advs.202502032 (PMC12442624; doi:10.1002/advs.202502032)
Supplement: Supplementary file 1 — Supporting Information [file ADVS-12-e02032-s002.pdf]

ADVANCED SCIENCE
Open Access

Supporting Information

for *Adv. Sci.*, DOI 10.1002/advs.202502032

Activation of Kir4.1 Channels by 2-D08 Promotes Myelin Repair in Multiple Sclerosis

Mingdong Liu, Shengyu Jin, Xin Fu, Chong Xie, Yi Chen, Liangtang Chang, Yongheng Fan, Donghua He, Xiaoqi Hong, Xi Shen, Xiaoli Zheng, Qiyue Wang, Dao Shi, Fangyuan Li, Daishun Ling, Yangtai Guan, Neng Gong* and Xiaoping Tong**

Supplementary information includes:
Supplementary figures 1-19 and figure legends, and 3 supplementary movie legends

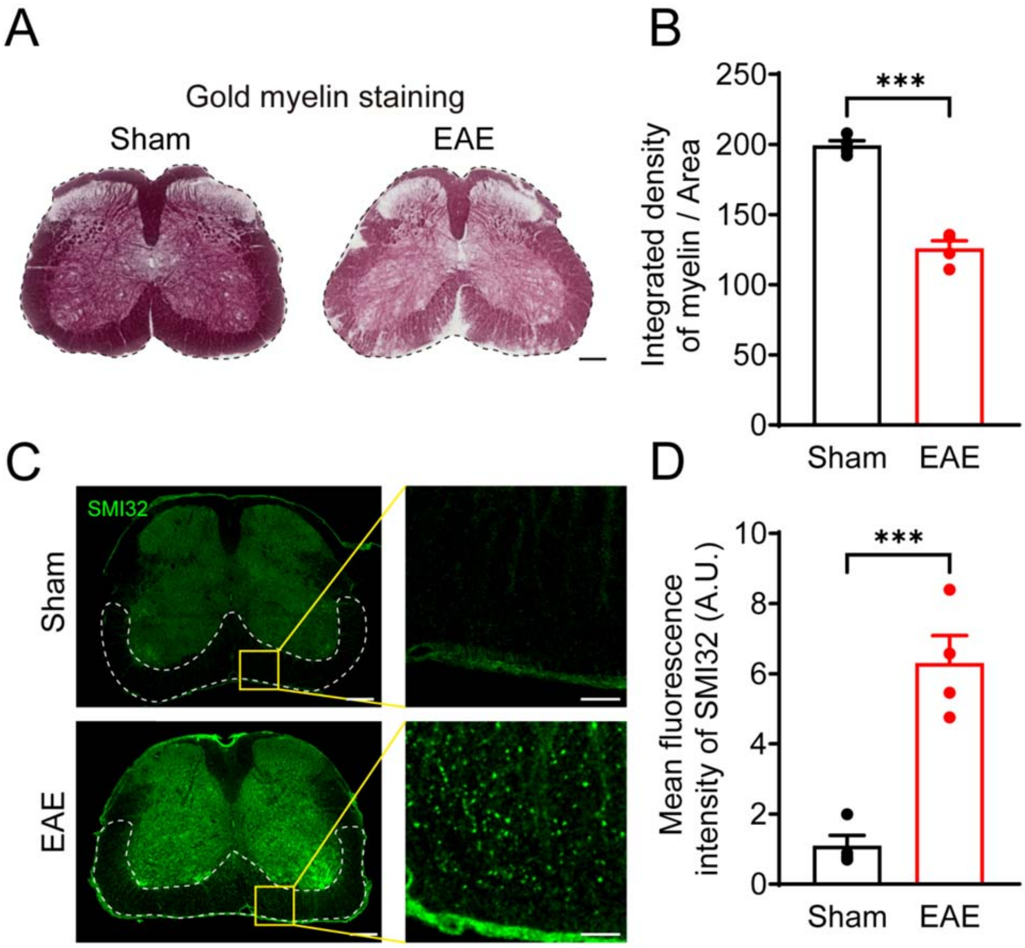

Fig. S1 | Characterization of myelin integrity and axonal damage in EAE mice.
(A) Representative images of gold myelin staining in spinal cord from Sham and EAE mice. Scale bars, 200 μm.
(B) Quantification of myelin integrity, expressed as integrated density of myelin per area, n = 4 mice for each group, two-tailed unpaired t-test, ***p < 0.001.
(C) Representative immunofluorescence images of SMI32 staining in the spinal cord from Sham and EAE mice. Scale bars, 200 μm (left panel) and 50 μm (right panel).
(D) Quantification of mean fluorescence intensity of SMI32, n = 4 mice for each group, two-tailed unpaired t-test, ***p < 0.001.

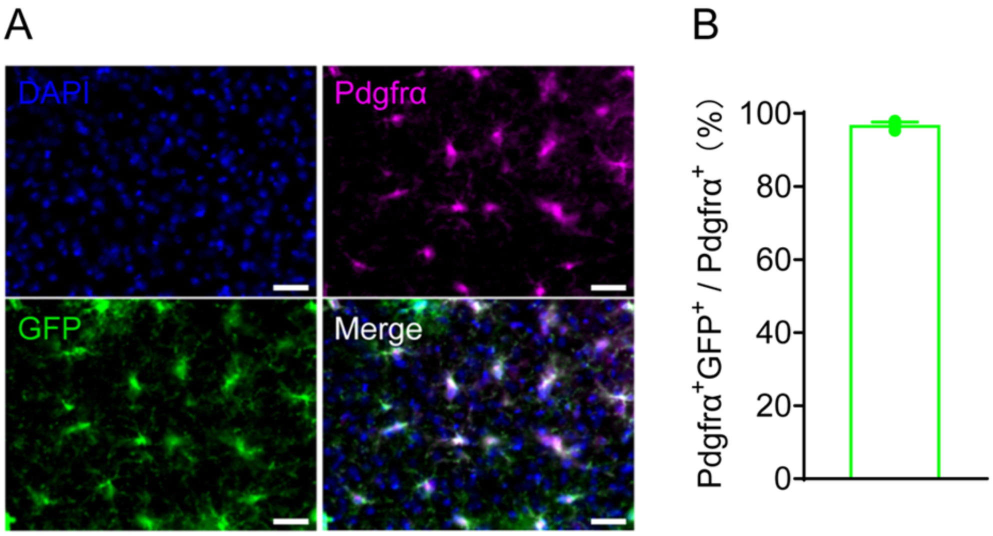

Fig. S2 | The specificity of OPC expression in ventral spinal cord of Pdgfrα-creER^{TM}; mGFP transgenic mouse strain.

(A) Representative images of immunofluorescence staining show colocalization between GFP-labeled cells and Pdgfrα antibody. Scale bars, 20 μm.

(B) Bar graph illustrated the mean percentage of GFP-positive cells in Pdgfrα-creER^{TM}; mGFP mice labeled with Pdgfrα antibody, n = 3 mice.

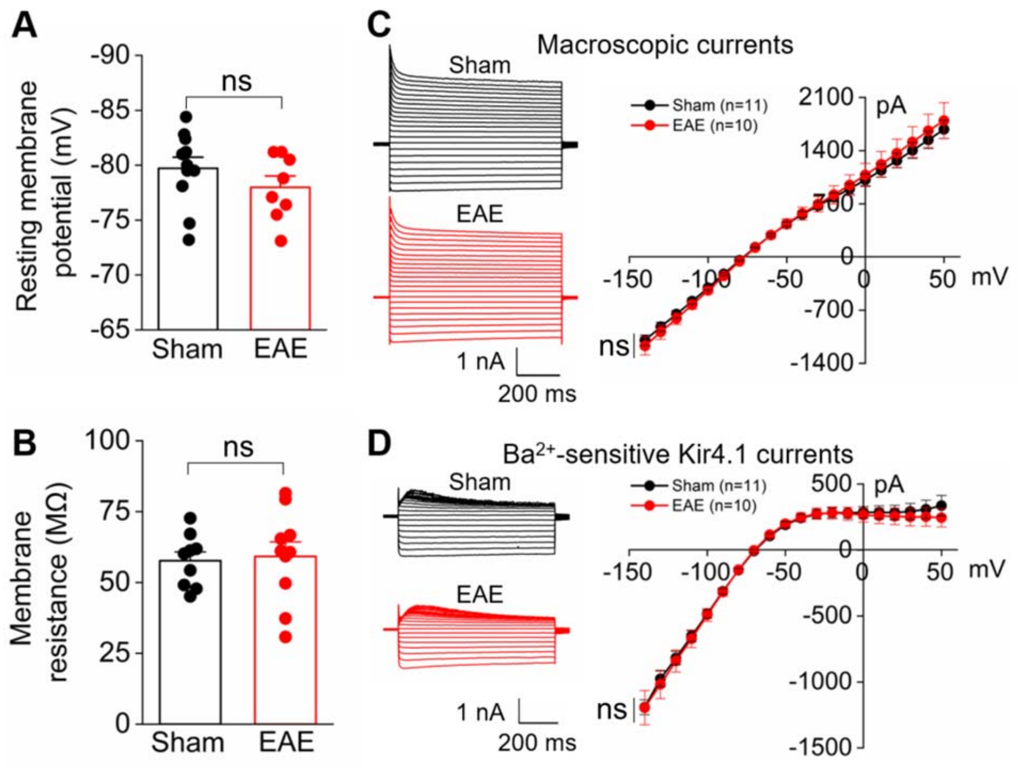

Fig. S3 | No change of Kir4.1 currents in dorsal spinal cord OPCs of EAE mice.

(A) Quantification of resting membrane potentials (RMPs) in dorsal spinal cord OPCs from sham and EAE mice. n = 11 cells from 5 sham mice and n = 9 cells from 4 EAE mice, two-tailed unpaired t-test, ns: not significant.

(B) Quantification of membrane resistances in dorsal spinal cord OPCs from sham and EAE mice. n = 11 cells from 5 sham mice and n = 9 cells from 4 EAE mice, two-tailed unpaired t-test, ns: not significant.

(C) Left: Representative traces of macroscopic K^+ currents in dorsal spinal cord OPCs from sham (black) and EAE (red) mice. Right: Average I/V plot. n = 11 cells from 5 sham mice and n = 10 cells from 4 EAE mice, two-tailed unpaired t-test, ns: not significant.

(D) Left: Representative traces of Ba^{2+}-sensitive Kir4.1 currents in dorsal spinal cord OPCs from sham (black) and EAE (red) mice. Right: Average I/V plot. n = 11 cells from 5 sham mice and n = 10 cells from 4 EAE mice, two-tailed unpaired t-test, ns: not significant.

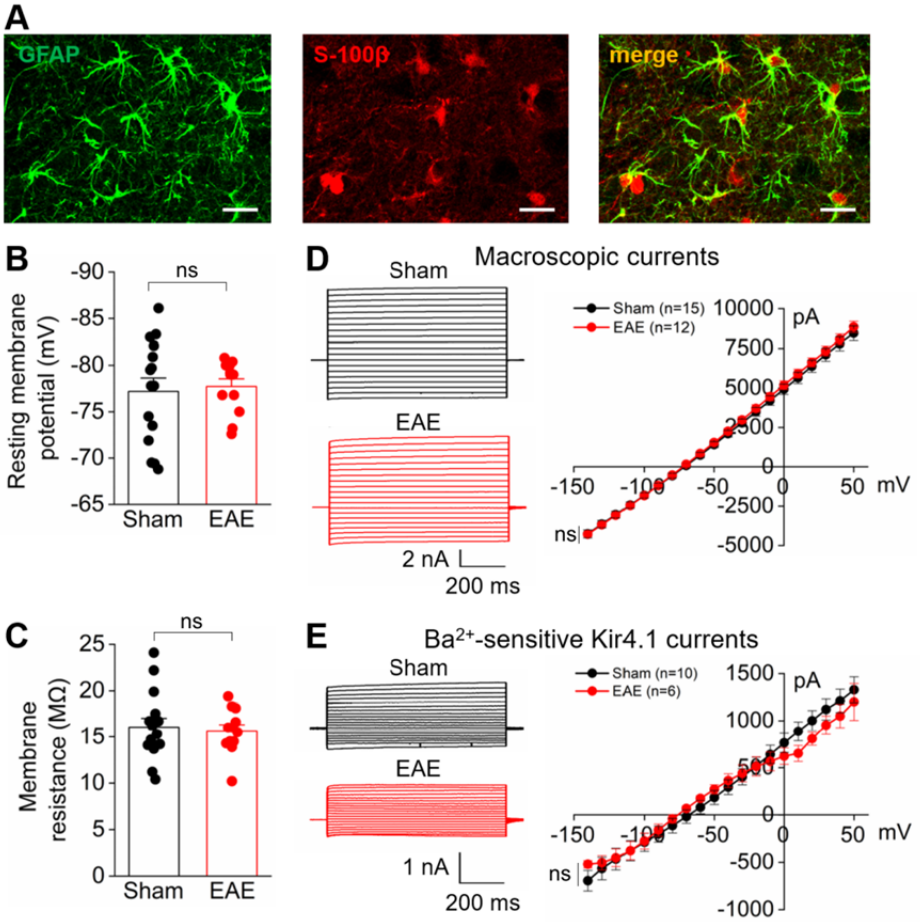

Fig. S4 | No change of Kir4.1 currents in ventral spinal cord astrocytes of EAE mice.

(A) Representative immunofluorescence images of GFAP (green) and S-100β (red) labeling in astrocytes. Scale bars, 20 μm.

(B) Quantification of resting membrane potentials (RMPs) in ventral spinal cord astrocytes from sham and EAE mice, n = 15 cells from 5 sham and n = 12 cells from 5 EAE mice, two-tailed unpaired t-test, ns: not significant.

(C) Quantification of membrane resistances in ventral spinal cord astrocytes from sham and EAE mice. n = 15 cells from 5 sham and n = 12 cells from 5 EAE mice, two-tailed unpaired t-test, ns: not significant.

(D) Representative traces (left) and average I/V curves (right) of macroscopic K^+ currents in ventral spinal cord astrocytes from sham (black) and EAE (red) mice. n = 15 cells from 5 sham and n = 12 cells from 5 EAE mice, two-tailed unpaired t-test, ns: not significant.

(E) Representative traces (left) and average I/V curves (right) of Ba^{2+}-sensitive Kir4.1 currents in ventral spinal cord astrocytes from sham (black) and EAE (red) mice. n = 10 cells from 5 sham and n = 6 cells from 5 EAE mice, two-tailed unpaired t-test, ns: not significant.

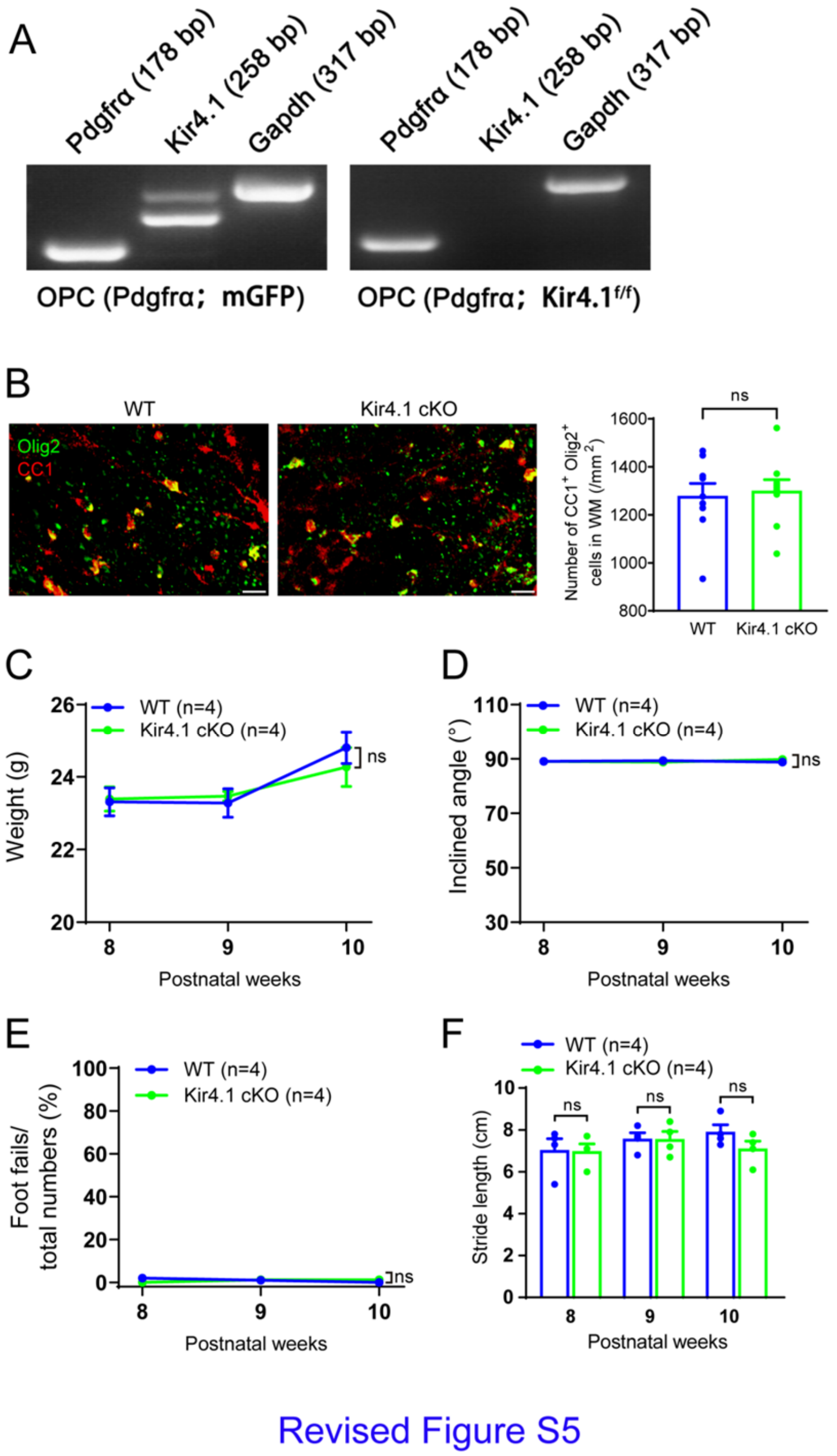

Fig. S5 | Kir4.1 conditional knockout in OPCs and its functional consequences.

(A) RT-PCR analysis of Pdgfra, Kir4.1 and Gapdh in OPCs isolated from Pdgfra; mGFP and Pdgfra; Kir4.1$^{f/f}$ mice, confirming targeted deletion of Kir4.1 in OPCs.

(B) Representative images of CC1^{+}/Olig2^{+}-labeled mature oligodendrocytes in the spinal cord of WT and Kir4.1 cKO adult mice. Bar graph showed the quantitative

statistical analysis of the colocalization of CC1 (red) and Olig2 (green) staining. n = 3 mice for each group, two-tailed unpaired t-test, ns: not significant. Scale bars, 20 μm.
(C) Body weight measurements of WT and Kir4.1 cKO adult mice. n = 4 mice for each group, two-tailed unpaired t-test, ns: not significant.
(D, E) Inclined angle (D) and grid walking task (E) measurements in WT and Kir4.1 cKO adult mice. n = 4 mice for each group, two-tailed unpaired t-test, ns: not significant.
(F) Summary of stride length in WT and Kir4.1 cKO adult mice. n = 4 mice for each group, two-tailed unpaired t-test, ns: not significant.

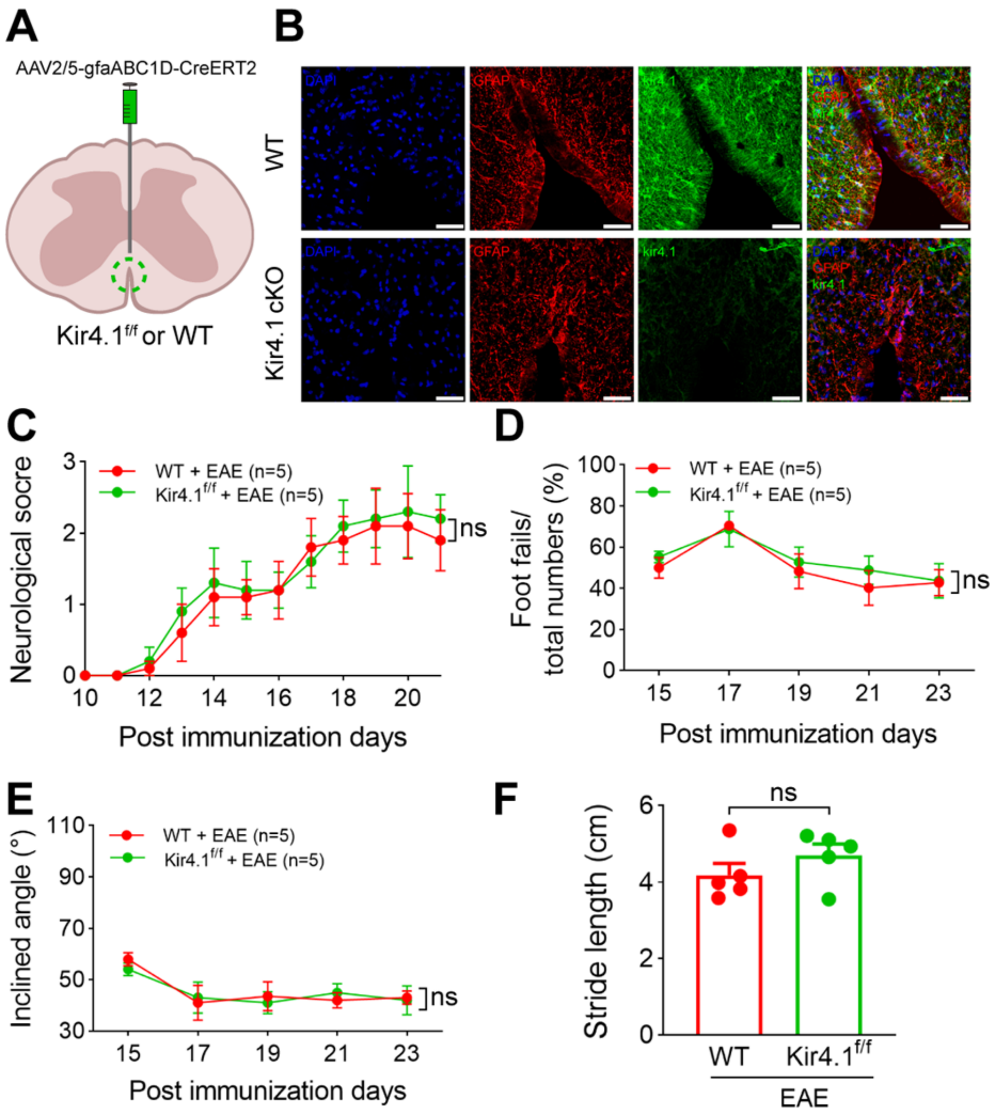

Fig. S6 | Kir4.1 knockout in astrocytes did not result in more severe pathological symptoms of EAE mice.

(A) Schematic illustration of the experimental timeline for AAV injection into the ventral spinal cord of WT or Kir4.1$^{f/f}$ mice prior to EAE induction.

(B) Representative immunofluorescence images showing colocalization of GFAP (red) and Kir4.1 (green) in astrocytes. Notably, Kir4.1 immunofluorescence was significantly reduced in Kir4.1$^{f/f}$ mice. Scale bars, 50 μm.

(C) Neurological scores of WT EAE and Kir4.1 cKO EAE mice, n = 5 mice for each group, two-tailed unpaired t-test, ns: not significant.

(D, E) Grid walking and inclined angle performance in WT EAE and Kir4.1 cKO EAE mice, n = 5 mice for each group, two-tailed unpaired t-test, ns: not significant.

(F) Summary of stride length in WT EAE and Kir4.1 cKO EAE mice, n = 5 mice for each group, two-tailed unpaired t-test, ns: not significant.

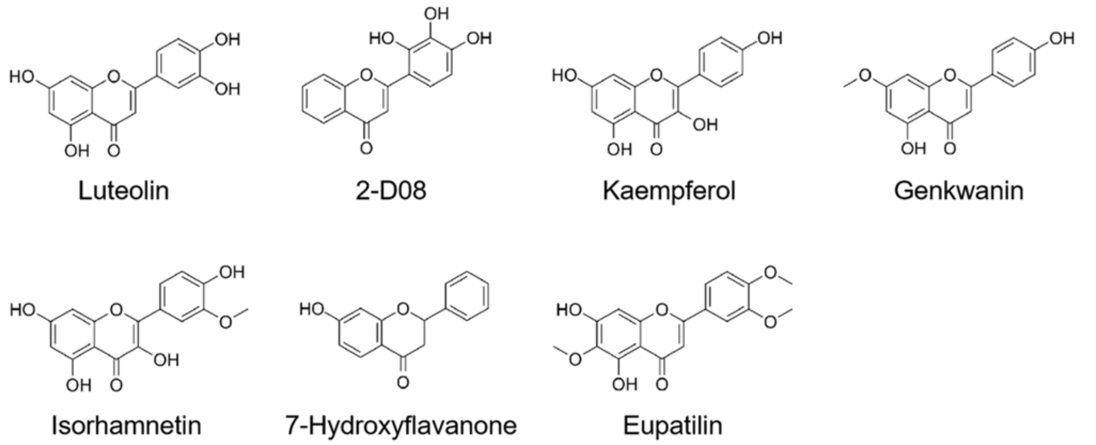

Fig. S7 | Chemical structures of luteolin and its analogues.

Chemical structures of luteolin, 2-D08, kaempferol, genkwanin, isorhamnetin, 7-hydroxyflavanone, and eupatilin, respectively.

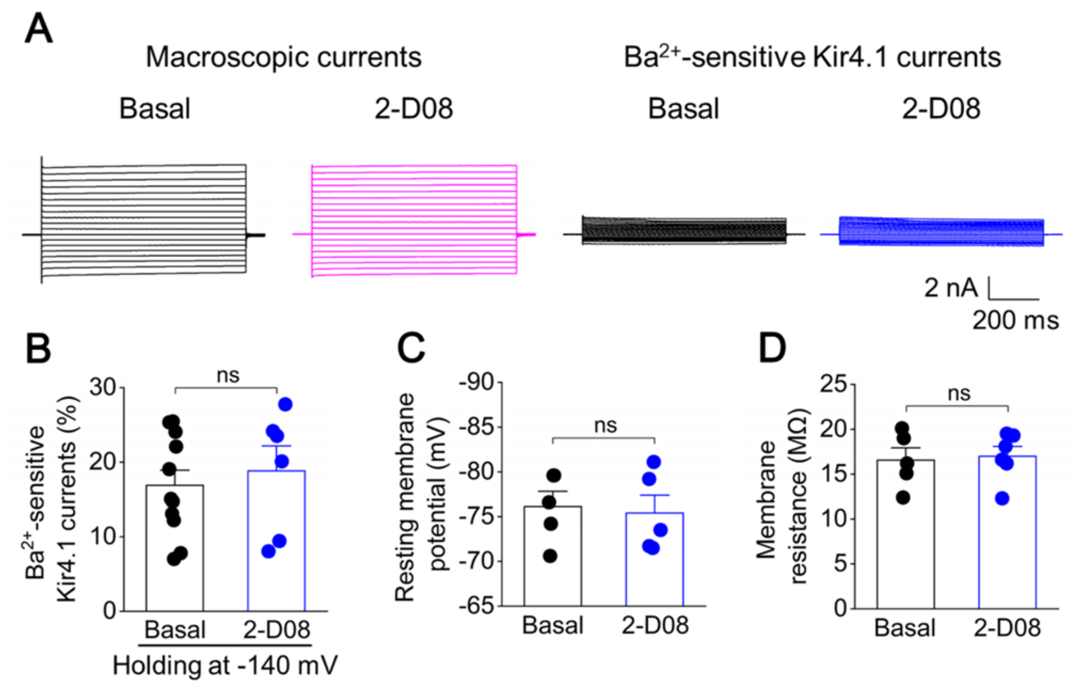

Fig. S8 | 2-D08 had no obvious effect on Ki4.1 channels in astrocytes.

(A) Left: Representative traces of macroscopic K^+ currents in ventral spinal cord astrocytes under basal conditions (black) and after 2-D08 treatment (magenta). Right: Representative traces of Ba^{2+}-sensitive Kir4.1 currents in ventral spinal cord astrocytes under basal conditions (black) and after 2-D08 treatment (blue).

(B) Quantification of the percentage increase in Ba^{2+}-sensitive Kir4.1 currents in ventral spinal cord astrocytes under basal conditions and after 2-D08 treatment. n = 11 cells for basal condition and n = 6 cells for 2-D08 group, two-tailed unpaired t-test, ns: not significant.

(C, D) Quantifications of resting membrane potentials (RMPs) (C) and membrane resistances (D) in ventral spinal cord astrocytes under basal conditions and after 2-D08 treatment. For RMP, n = 5 and 5 cells for basal condition and 2-D08 group, two-tailed unpaired t-test, ns: not significant. For membrane resistance, n = 5 and 6 cells for basal condition and 2-D08 group, respectively, two-tailed unpaired t-test, ns: not significant.

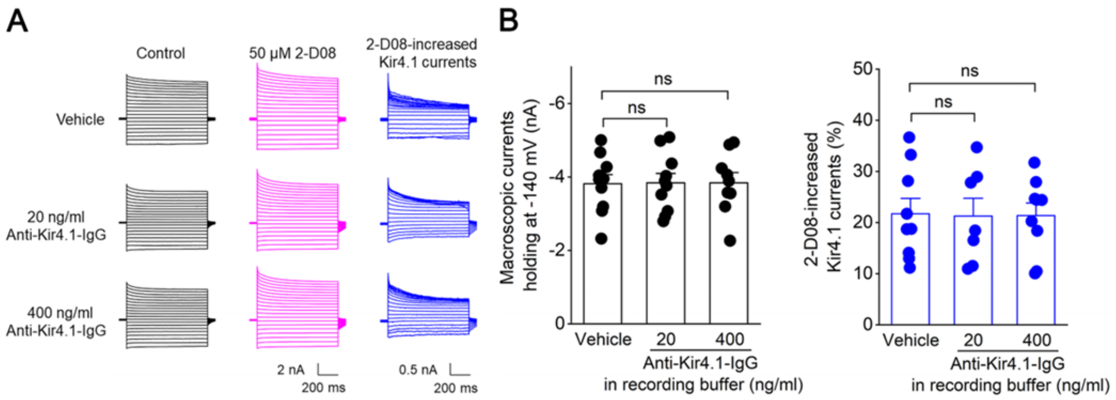

Fig. S9 | Anti-Kir4.1-IgG did not affect the Kir4.1 channel activity.

(A) Representative traces showed the macroscopic currents of Kir4.1-mCherry transfected HEK-293T cells in the basal control (black) and after 2-D08 applications (magenta) in the presence of vehicle, 20 ng/ml anti-Kir4.1-IgG, and 400 ng/ml anti-Kir4.1-IgG in the recording buffer. HEK-293T cells were incubated with anti-Kir4.1-IgG for 1 hour before patch-clamp recording.

(B) Bar graphs showed the total macroscopic currents (left panel) and the percentage of 2-D08-increased Kir4.1 channel currents (right panel) in the presence of vehicle, 20 ng/ml anti-Kir4.1-IgG, and 400 ng/ml anti-Kir4.1-IgG in the recording buffer when the cell voltage was held at -140 mV. For macroscopic currents analysis, n = 10, 10 and 9 cells for vehicle, 20 ng/ml anti-Kir4.1-IgG, 400 ng/ml anti-Kir4.1-IgG, one-way ANOVA with Tukey-Kramer multiple comparisons test, ns: not significant. For Kir4.1 channel currents analysis, n = 9, 7 and 9 cells for vehicle, 20 ng/ml anti-Kir4.1-IgG, 400 ng/ml anti-Kir4.1-IgG, respectively, one-way ANOVA with Tukey-Kramer multiple comparisons test, ns: not significant.

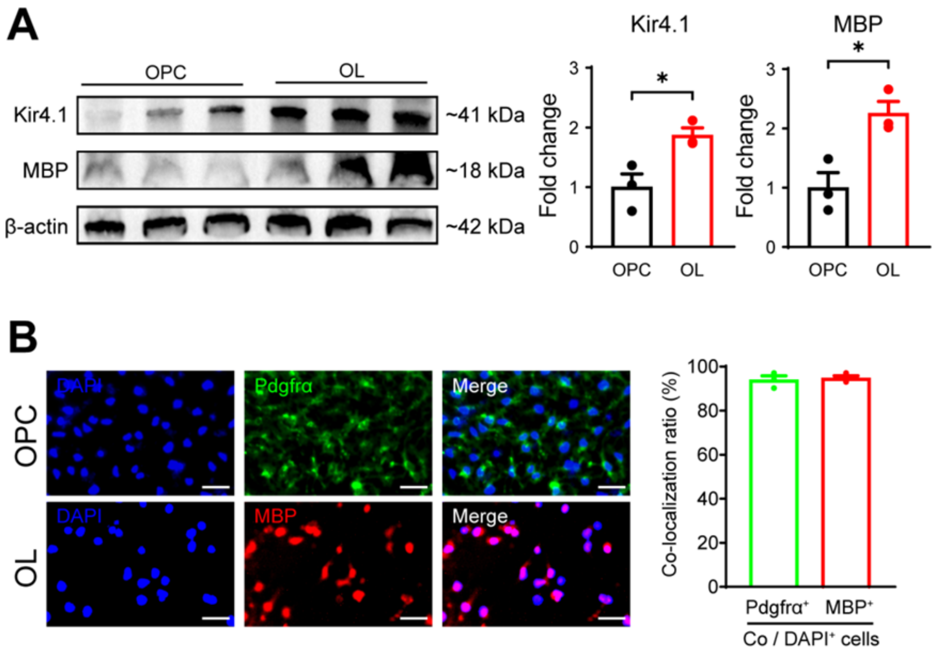

Fig. S10 | Expression profiling of Kir4.1 and MBP in oligodendrocyte lineage cells.

(A) Western blotting images (left panel) and the statistical analysis (right panel) of Kir4.1 and MBP expression in both primary cultured OPCs and their differentiated mature oligodendrocytes (OLs). n = 3 independent replicate samples for each group, two-tailed unpaired t-test, $^*p < 0.05$.

(B) Representative images of immunofluorescence staining of Pdgfra and MBP in primary cultured OPCs and their differentiated mature oligodendrocytes (OLs). The co-localization ratio quantifies the percentage of Pdgfra$^+$ OPCs or MBP$^+$ OLs among the total DAPI-labeled cells. n = 3 independent replicate samples for culturing. Scale bars, 20 μm.

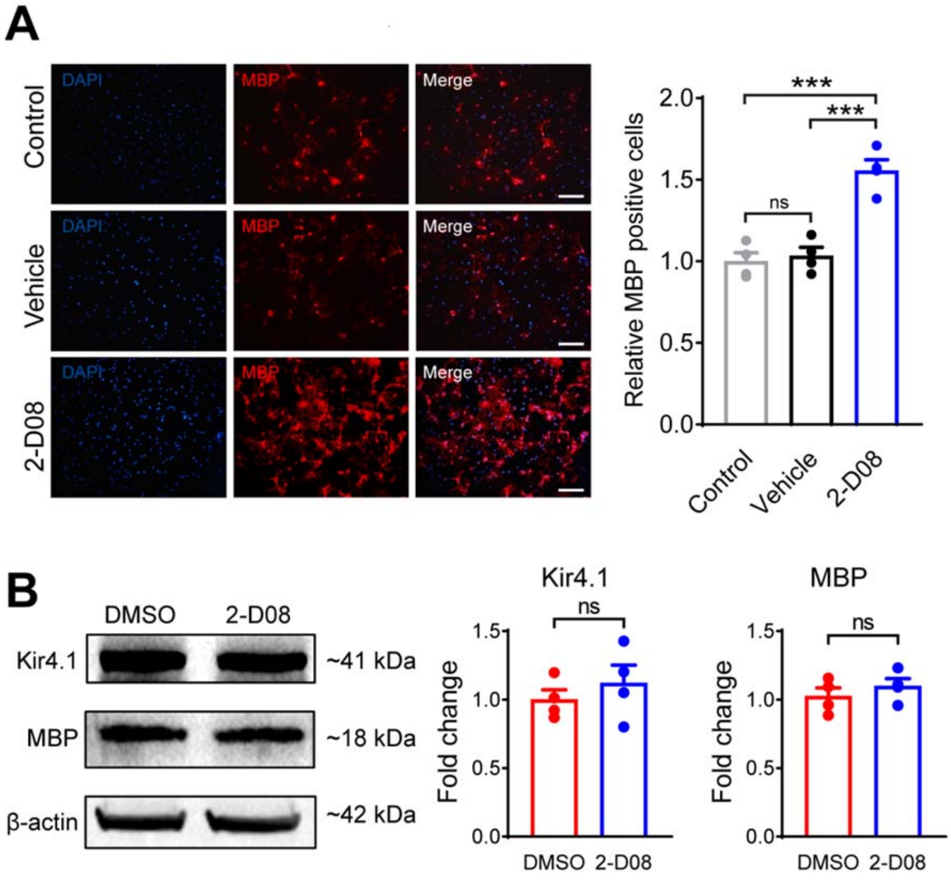

Fig. S11 | Effects of 2-D08 treatment on myelination and Kir4.1 expression *in vitro.*

(A) Representative images of immunofluorescence staining of MBP after the primary OPCs cultured for 72 hours under control, vehicle, and 2-D08 treatment *in vitro*. n = 3 independent replicate samples for each group, one-way ANOVA with Tukey-Kramer multiple comparisons, ***$p < 0.001$, ns: not significant. Scale bars, 50 μm.

(B) Western blotting analysis of Kir4.1 and MBP in OLs from DMSO-treated, and 2-D08-treated cultures. Quantification of protein levels of Kir4.1 and MBP in OL cell culture, n = 4 independent replicate samples for each group, two-tailed unpaired t-test, ns: not significant.

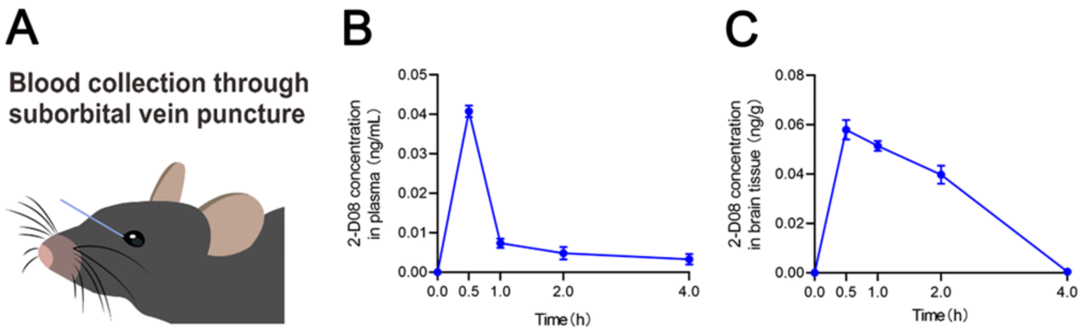

Fig. S12 | Plasma and brain tissue concentrations of 2-D08 after intraperitoneal injection in mice.

(a) The cartoon illustrated blood sample collection (0.1 mL) via vein puncture after intraperitoneal (i.p.) injection of 2-D08 (1 mg/kg) in mice.

(b) High-performance liquid chromatography (HPLC) analysis depicted plasma concentrations of 2-D08 at 0, 0.5, 1, 2, and 4 hours post-injection. n = 3 mice.

(c) HPLC analysis revealed that 2-D08 penetrated the blood-brain barrier (BBB) and accumulated in the brain region. n = 3 mice.

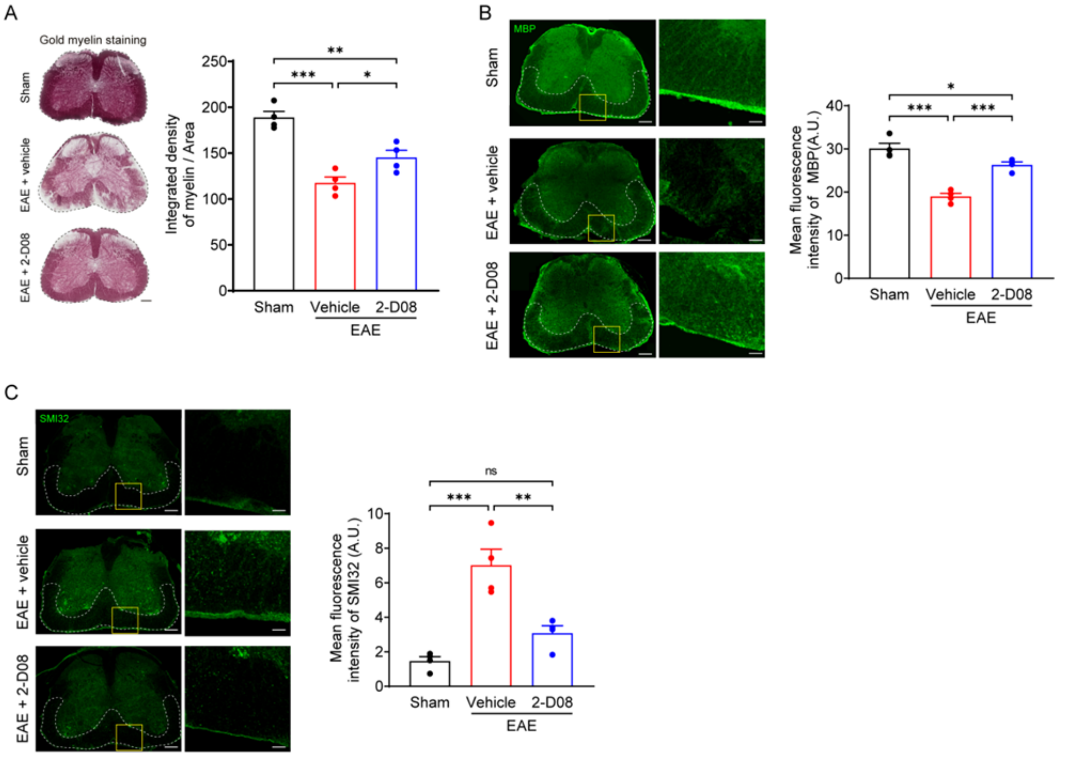

Fig. S13 | Effect of 2-D08 on myelin integrity and neuronal markers in EAE mice.

(A) Representative images and quantification of myelin integrity of gold myelin staining in spinal cord from Sham, EAE + vehicle, and EAE + 2-D08 mice. n = 4 mice for each group, one-way ANOVA with Tukey-Kramer multiple comparisons, $*p < 0.05$, $**p < 0.01$, $***p < 0.001$. Scale bar, 200 μm.

(B) Representative images of immunofluorescence staining of MBP in spinal cord from Sham, EAE + vehicle, and EAE + 2-D08 mice. Scale bars: 200 μm (left panels) and 50 μm for the magnified images in the yellow squares. Bar graph showed the quantification of mean fluorescence intensity of MBP. n = 4 mice for each group, one-way ANOVA with Tukey-Kramer multiple comparisons, $*p < 0.05$, $***p < 0.001$.

(C) Representative images of immunofluorescence staining of SMI32 in spinal cord from Sham, EAE + vehicle, and EAE + 2-D08 mice. Scale bars: 200 μm (left panels) and 50 μm for the magnified images in the yellow squares. Bar graph showed the quantification of mean fluorescence intensity of SMI32. n = 4 mice for each group, one-way ANOVA with Tukey-Kramer multiple comparisons, $**p < 0.01$, $***p < 0.001$, ns: not significant.

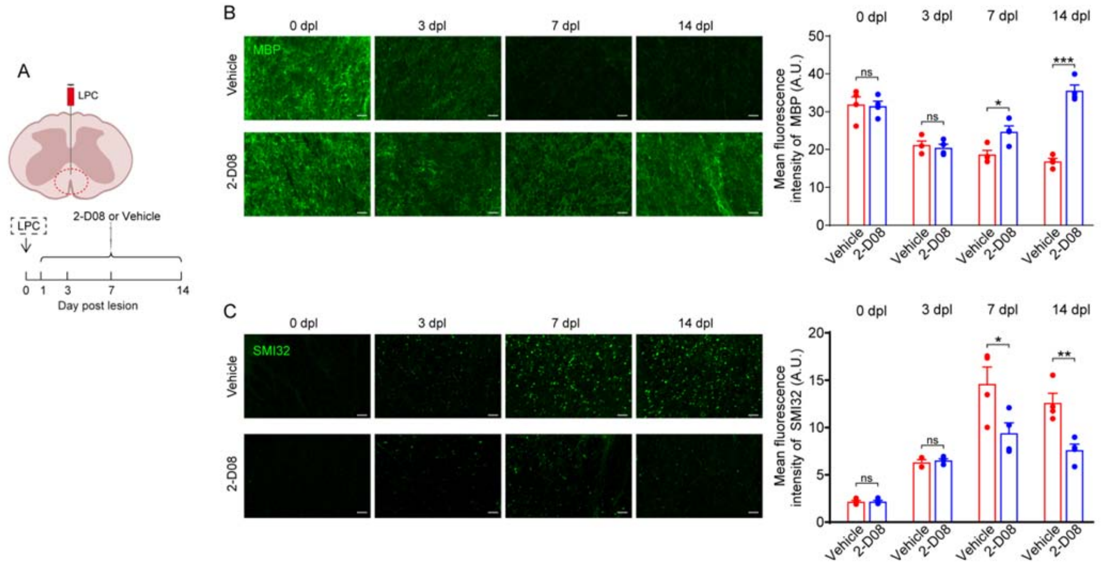

Fig. S14 | Temporal effects of 2-D08 treatment on myelin repair and axonal integrity following lysolecithin-induced demyelination.

(A) Experimental timeline of 2-D08 or vehicle administration in lysolecithin (LPC)-induced demyelination mouse model. dpl: days post lesion.

(B) Representative images of immunofluorescence staining of MBP in spinal cord from vehicle-treated or 2-D08-treated mice at 0, 3, 7, and 14 dpl follow LPC-injections. The bar graph on the right showed the quantification of mean fluorescence intensity of MBP. n = 4 mice for each group, two-tailed unpaired t-test, *$p < 0.05$, ***$p < 0.001$, ns: not significant. Scale bars, 20 μm.

(C) Representative images of immunofluorescence staining of SMI32 in spinal cord from vehicle-treated or 2-D08-treated mice at 0, 3, 7, and 14 dpl follow LPC-injections. The bar graph on the right showed the quantification of mean fluorescence intensity of SMI32. n = 4 mice for each group, two-tailed unpaired t-test, *$p < 0.05$, **$p < 0.01$, ns: not significant. Scale bars, 20 μm.

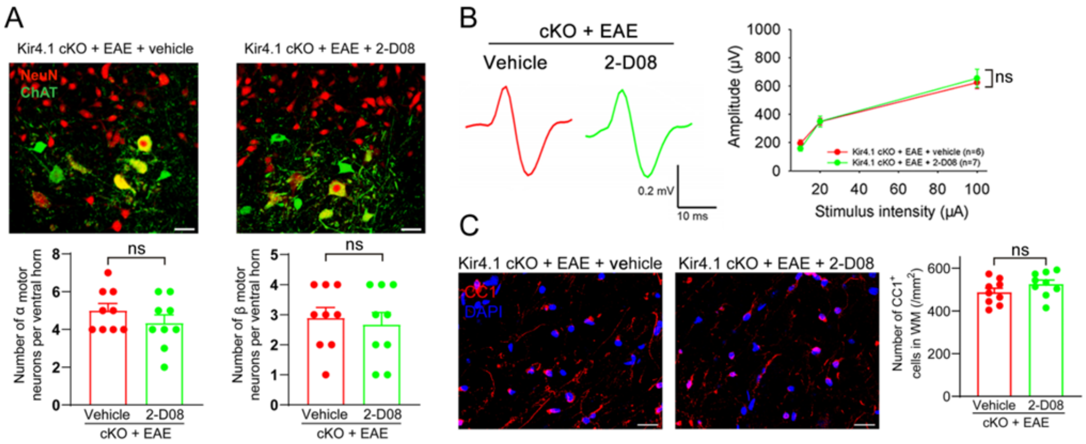

Fig. S15 | 2-D08 did not promote the motor neuron functional recovery after Kir4.1 deletion in OPCs.

(A) Representative images of immunofluorescence staining of motor neurons labeled for NeuN (red) and ChAT (green) in the spinal cord ventral horn from Kir4.1 cKO + EAE + vehicle and Kir4.1 cKO + EAE + 2-D08 groups. Quantification of number of α motor neurons (left) and β motor neurons (right). n = 9 spinal cord slices from 3 mice for each group, two-tailed unpaired t-test, ns: not significant. Scale bars: 50 μm.

(B) Representative sample recordings of MEPs from Kir4.1 cKO + EAE + vehicle and Kir4.1 cKO + EAE + 2-D08 groups (left panel). Summary graph of MEP amplitudes from Kir4.1 cKO + EAE + vehicle and Kir4.1 cKO + EAE + 2-D08 groups (right panel), n = 6 mice for Kir4.1 cKO + EAE + vehicle groups and 7 mice for Kir4.1 cKO + EAE + 2-D08 groups. For MEP amplitude at stimulus intensity of 10 μA and 20 μA, two-tailed unpaired t-test, ns: not significant. For MEP amplitude at stimulus intensity of 100 μA, two-tailed Mann-Whitney test, ns: not significant.

(C) Representative images of immunofluorescence staining of CC1 in spinal cord slices from Kir4.1 cKO + EAE + vehicle and Kir4.1 cKO + EAE + 2-D08 groups. Bar graph showed the quantification of the number of CC1-positive cells in each condition. n = 9 spinal cord slices from 3 mice per group, two-tailed unpaired t-test, ns: not significant. Scale bars: 20 μm.

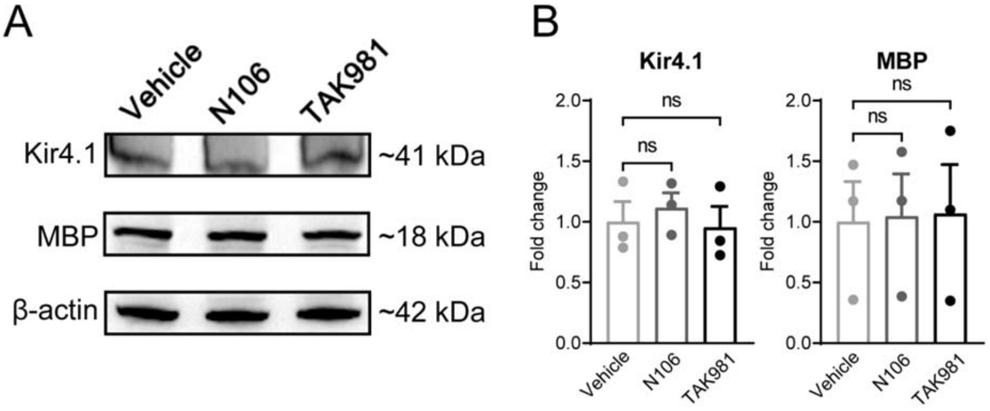

Fig. S16 | SUMOylation pathway did not change the Kir4.1 and MBP expression during OLs maturation.

(A) Representative western blotting images of Kir4.1 and MBP expression levels in OLs derived from primary OPC cultures treated with vehicle, N106 (SUMOylation activator), or TAK981 (SUMOylation inhibitor).

(B) Bar graphs showed the quantification of Kir4.1 and MBP protein levels during OLs maturation derived from primary OPCs. n = 3 independent replicate samples per group, two-tailed unpaired t-tests were performed between vehicle and N106, and vehicle and TAK981 groups. ns: not significant.

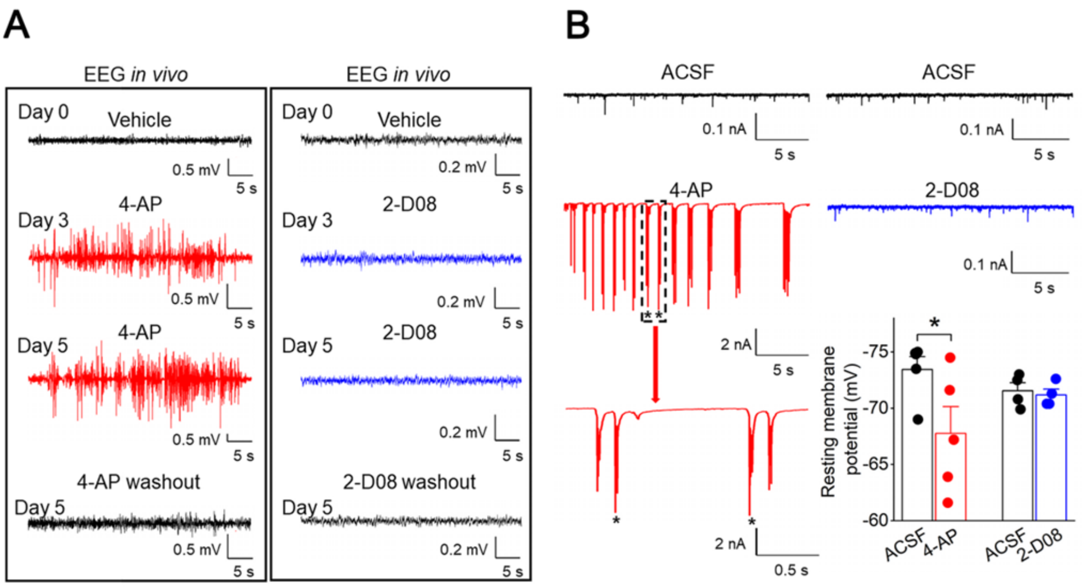

Fig. S17 | Paroxysmal seizures were not observed in 2-D08-treated mice.

(A) Representative traces showed the occurrence of focal seizures in a mouse on days 3 and day 5 after 4-AP and 2-D08 injections (i.p., left panel). There were no paroxysmal alterations in 2-D08-treated mice (i.p., right panel). n = 2 mice for each 4-AP and 2-D08 treated groups.

(B) Representative traces recorded from neocortical L5 pyramidal neurons. Asterisks indicated the events shown below at enlarged timescale. Quantitative summary of the RMPs from whole-cell patch clamp recordings, n = 5 cells for 4-AP and 4 cells for 2-D08 group, two-tailed paired t-test, *$p < 0.05$.

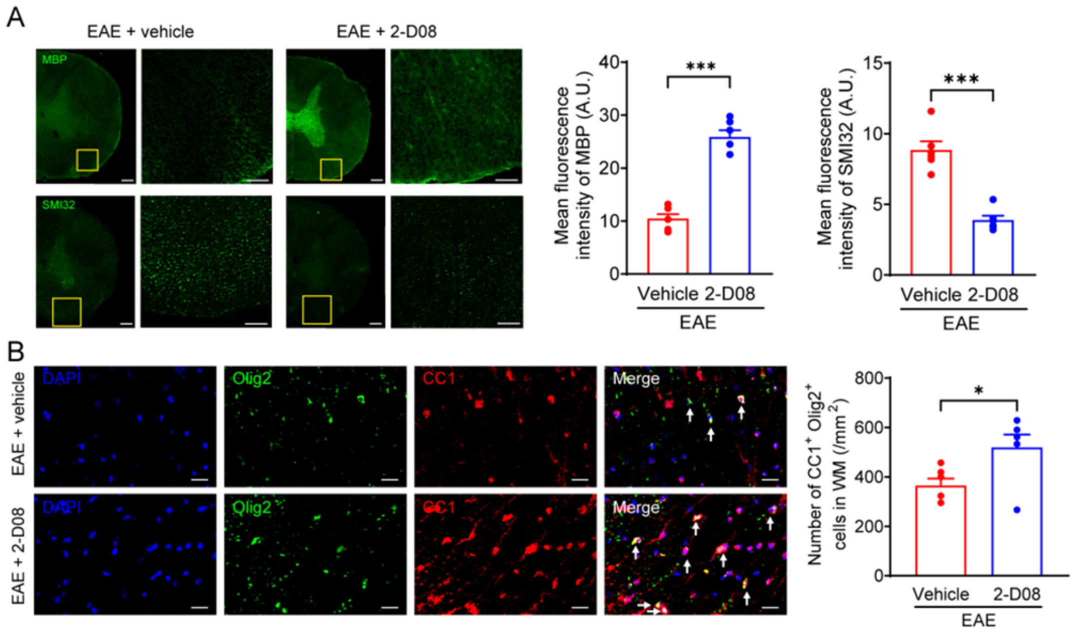

Fig. S18 | Effects of 2-D08 treatment on myelin repair in EAE marmosets.

(A) Representative images of immunofluorescence staining of MBP and SMI32 in spinal cord from vehicle- and 2-D08-treated EAE marmosets. Bar graphs showed the quantifications of mean fluorescence intensity of MBP and SMI32. n = 6 spinal cord slices from 2 marmosets per group, two-tailed unpaired t-test, ***p < 0.001. Scale bars: 200 μm (left panels) and 50 μm for the magnified images in the yellow squares.

(B) Representative images of $CC1^+/Olig2^+$-labeled mature oligodendrocytes in the spinal cord from vehicle- and 2-D08-treated EAE marmosets. The bar graph showed the quantification of the colocalization of CC1 and Olig2 in the white matter of the spinal cord. n = 6 spinal cord slices from 2 marmosets per group, two-tailed unpaired t-test, *p < 0.05. Scale bars, 20 μm.

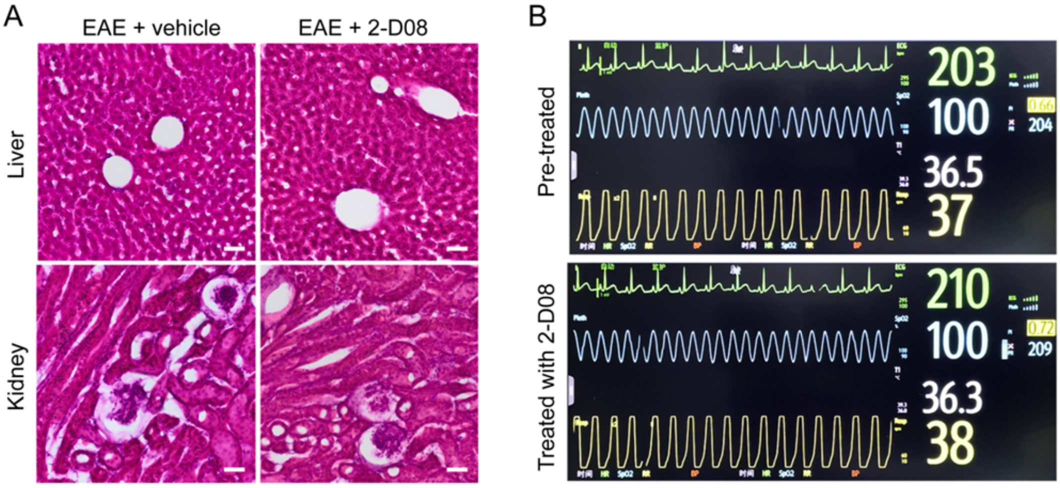

Fig. S19 | 2-D08 treatment did not cause obvious peripheral or cardiac toxicity.

(A) Representative hematoxylin-eosin (HE) staining images from sham and 2-D08-treated EAE mice. Scale bars: 50 µm.

(B) The electrocardiogram (ECG) recordings from EAE marmosets were monitored for at least 1 hour following 2-D08 administration (1 mg/kg, intraperitoneal injection).

Supplementary movie legends

movie S1: Representative movie of the jumping performance of marmoset 150121 before the induction of EAE with $rhMOG_{1\text{-}125}$ at day 0 post immunization.

movie S2: Representative movie of the jumping performance of marmoset 150121 at the onset of EAE symptoms at day 40 post immunization.

movie S3: Representative movie of the jumping performance of marmoset 150121 treated with 2-D08 at day 70 post immunization.
