# Supplementary material for: Activation of Kir4.1 Channels by 2‐D08 Promotes Myelin Repair in Multiple Sclerosis
Source: Adv Sci (Weinh). 2025 Jun 5;12(34):e02032. doi: 10.1002/advs.202502032 (PMC12442624; doi:10.1002/advs.202502032)
Supplement: Supplementary file 2 — Supporting Information [file ADVS-12-e02032-s006.pdf]

ADVANCED SCIENCE
Open Access

Supporting Information

for *Adv. Sci.*, DOI 10.1002/advs.202502032

Activation of Kir4.1 Channels by 2-D08 Promotes Myelin Repair in Multiple Sclerosis

Mingdong Liu, Shengyu Jin, Xin Fu, Chong Xie, Yi Chen, Liangtang Chang, Yongheng Fan, Donghua He, Xiaoqi Hong, Xi Shen, Xiaoli Zheng, Qiyue Wang, Dao Shi, Fangyuan Li, Daishun Ling, Yangtai Guan, Neng Gong* and Xiaoping Tong**

Table S1 Information of human serum samples

Health#	Age	Sex	Disease
1	63	F	NO
2	51	F	NO
3	40	M	NO
4	37	F	NO
5	36	F	NO
6	32	F	NO
**Patient#**	**Age**	**Sex**	**Disease diagnosis**
1	70	F	MS
2	69	F	MS
3	61	M	MS
4	56	F	MS
5	40	F	MS
6	33	F	MS
7	32	M	MS
8	25	F	MS
9	23	F	MS
