# Supplementary material for: Activation of Kir4.1 Channels by 2‐D08 Promotes Myelin Repair in Multiple Sclerosis
Source: Adv Sci (Weinh). 2025 Jun 5;12(34):e02032. doi: 10.1002/advs.202502032 (PMC12442624; doi:10.1002/advs.202502032)
Supplement: Supplementary file 3 — Supporting Information [file ADVS-12-e02032-s004.pdf]

ADVANCED SCIENCE
Open Access

Supporting Information

for *Adv. Sci.*, DOI 10.1002/advs.202502032

Activation of Kir4.1 Channels by 2-D08 Promotes Myelin Repair in Multiple Sclerosis

Mingdong Liu, Shengyu Jin, Xin Fu, Chong Xie, Yi Chen, Liangtang Chang, Yongheng Fan, Donghua He, Xiaoqi Hong, Xi Shen, Xiaoli Zheng, Qiyue Wang, Dao Shi, Fangyuan Li, Daishun Ling, Yangtai Guan, Neng Gong* and Xiaoping Tong**

Table S2: A list of the used reagents

REAGENT or RESOURCE	SOURCE	IDENTIFIER
Antibodies		
Rabbit anti-NG2	Millipore	Cat# AB5320; RRID: AB_11213678
Chicken anti-GFAP	Abcam	Cat# ab4674; RRID: AB_304558
Rabbit anti-Kir4.1	Alomone Labs	Cat# APC-035; RRID: AB_2040120
Mouse anti-APC (CC-1)	Millipore	Cat# OP80; RRID: AB_2057371
Goat anti-Pdgfrα	R&D Systems	Cat# AF1062; RRID: AB_2236897
Chicken anti-GFP	Abcam	Cat# AB13970; RRID: AB_300798
Mouse anti-Olig2	Millipore	Cat# MABN50 AB_10807410
Mouse anti-SMI32	Biolgend	AB_2564642
mouse antibody to mCherry	Abcam	ab125096; RRID: AB_11133266
Rabbit anti-S100β	Abcam	Cat# Ab52642; RRID: AB_882426
Goat anti-Choline Acetyltransferase (ChAT)	Millipore	Cat# AB144P; RRID: AB_2079751
Rabbit anti-NeuN	Cell Signaling Technology	Cat# 24307; RRID: AB_2651140
Mouse anti-Myelin Basic Protein (MBP)	Cell Signaling Technology	Cat# MAB382; RRID: AB_2073319
FYN	Cell Signaling Technology	Cat# 2109; RRID: AB_2106059

p-FYN	Cell Signaling Technology	Cat# 6943; RRID: AB_10013641
Mouse anti-β-actin Clone AC-15	Thermo Fisher Scientific	Cat# MA515739; RRID: AB_10979409
goat anti-mouse IgG, HRP conjugated	Cwbio	CW0102
goat anti-rabbit IgG, HRP conjugated	Cwbio	CW0103
Goat anti-Chicken Alexa Fluor 488	Thermo Fisher Scientific	Cat# A11039; RRID: AB_2534096
Donkey anti-Mouse Alexa Fluor 488	Molecular	Cat# A21202; RRID: AB_141607
Donkey anti-Mouse Alexa Fluor 568	Invitrogen	Cat# A10037; RRID: AB_2757558
Donkey anti-goat Alexa Fluor 488	Molecular	Cat# A11055; RRID: AB_2534102
Donkey anti-Rabbit Alexa Fluor 488	Invitrogen	Cat# A21206; RRID: AB_2535792
Donkey anti-Rabbit Alexa Fluor 568	Invitrogen	Cat# A10042; RRID: AB_2534017
Alexa Fluor® 647 picolyl azide	Assay Biotech	Cat# C10643; RRID: AB_10686339
Biological samples		
Serum of MS patients and healthy individuals	Renji Hospital, Shanghai Jiao Tong University School of Medicine, China.	
Chemicals, Peptides, and Recombinant Proteins		
Deoxyribonuclease I (DNase I)	Worthington Biochemical corporation	Cat# LS002139 CAS: 9003-98-9
BSA	Sigma-Aldrich	Cat# A1933; CAS: 9048-46-8

EDTA	Sigma-Aldrich	Cat# E9884;<br>CAS: 60-00-4
DMEM/F12	Corning-cellgro	Cat# 10-092-CVRC
DMEM	Corning-cellgro	Cat# 10-013-CVRC
Trypsin	LIFE TECHNOLOGIES	Cat# 25200072
Tamoxifen	ABCONE	Cat# T56488;<br>CAS: 10540-29-1
2-D08	Top Science https://www.targetmol.cn/compound/2-d08	Cat# T7379;<br>CAS: 144707-18-6
Triton X-100	Sigma-Aldrich	Cat# T8787;<br>CAS: 9036-19-5
4-AP	Millipore	Cat# 275875;<br>CAS:504-24-5
Donkey serum	Ruite Biotechnology	Cat# w9030-05;<br>CAS:1234-00-00
DAPI	Cell Signaling Technology	Cat# 4083S;<br>CAS:28718-90-3
rhMOG$_{1-125}$	CUSABIO	CSB-EP619083HU
Experimental Models: Cell Lines		
Human Embryonic Kidney (HEK) 293T cells	ATCC	CRL-3216
Experimental Models: Organisms/Strains		
Mouse: Pdgfrα-creER^{TM}	The Jackson Laboratory	Cat# 018280
Mouse: Kir4.1$^{f/f}$	The Jackson Laboratory	Cat# 026826
Mouse: Rosa26-mGFP	The Jackson Laboratory	Cat# 007676
Mouse: C57BL/6	Slac Laboratory Animal	Cat# 000664
Recombinant DNA		

pLVX-IRES-mCherry	Clonetech	Cat# 631237
pcDNA3.1-Kcnj10	This paper	N/A
Software and Algorithms		
ImageJ	NIH	RRID: SCR_003070
pCLAMP 10.5	Molecular Devices	RRID: SCR_011323
ClampFit 10.5	Molecular Devices	N/A
OriginPro 2018	Origin Lab Corporation	RRID: SCR_014212
Adobe illustrator 2018	Adobe Inc.	RRID: SCR_010279
GraphPad InStat 3	GraphPad Software	RRID: SCR_000306
Leica Application Suite X	Leica Microsystems Inc.	RRID: SCR_013673
Zeus	Bio-Signal Technologies	N/A
